# Fingolimod induces neuronal-specific gene expression with potential neuroprotective outcomes in maturing neuronal progenitor cells exposed to HIV

**DOI:** 10.1007/s13365-017-0571-7

**Published:** 2017-09-14

**Authors:** Rebeca Geffin, Ricardo Martinez, Alicia de las Pozas, Biju Issac, Micheline McCarthy

**Affiliations:** 1 Research Service, Bruce W. Carter Veterans Affairs Medical Center, 1201 NW 16th Street, Miami, FL 33125 USA; 2 Department of Neurology, University of Miami Miller School of Medicine, 1120 NW 14th St, Miami, FL 33136 USA; 3 Division of Bioinformatics, Biostatistics and Bioinformatics Core, Sylvester Comprehensive Cancer Center, University of Miami Miller School of Medicine, 1550 NW 10th Avenue, Fox Building, Suite 300, Miami, FL 33136 USA

**Keywords:** Human immunodeficiency virus, Fingolimod, Neuron, Gene expression microarray, Neuroprotection

## Abstract

Fingolimod (FTY720), a structural analogue of sphingosine, targets sphingosine-1-phosphate receptor signaling and is currently an immunomodulatory therapy for multiple sclerosis. Fingolimod accesses the central nervous system (CNS) where its active metabolite, fingolimod phosphate (FTY720-P), has pleotropic neuroprotective effects in an inflammatory microenvironment. To investigate potential neuronal-specific mechanisms of fingolimod neuroprotection, we cultured the human neuronal progenitor cell line, hNP1, in differentiation medium supplemented with HIV- or Mock-infected supernatants, with or without FTY720-P. Gene expression was investigated using microarray and functional genomics. FTY720-P treatment increased differentially expressed (DE) neuronal genes by 33% in HIV-exposed and 40% in Mock-exposed cultures. FTY720-P treatment broadened the functional profile of DE genes in HIV-exposed versus Mock-exposed neurons, including not only immune responses but also transcriptional regulation and cell differentiation, among others. FTY720-P treatment downregulated the gene for follistatin, the antagonist of activin signaling, in all culture conditions. FTY720-P treatment differentially affected both glycolysis-related and immune response genes in Mock- or HIV-exposed cultures, significantly upregulating 11 glycolysis-related genes in HIV-exposed neurons. FTY720-P treatment also differentially upregulated genes related to innate immune responses and antigen presentation in Mock-exposed and more so in HIV-exposed neurons. However, in HIV-exposed neurons, FTY720-P depressed the magnitude of differential expression in almost half the genes, suggesting an anti-inflammatory potential. Moreover, in HIV-exposed neurons, FTY720-P reduced expression of the amyloid precursor protein (APP) gene, resulting in reduced expression of the APP protein. This study provides new evidence that fingolimod alters neuronal gene expression in inflammatory, viral-infected microenvironments, with the potential for neuroprotective effects.

**Electronic supplementary material:**

The online version of this article (doi:10.1007/s13365-017-0571-7) contains supplementary material, which is available to authorized users.

## Introduction

Fingolimod (FTY720, chemical name 2-amino-2[2-(4-octylphenyl)ethyl]-1-3-propanediol hydrochloride) is an analogue of sphingosine and is an FDA-approved oral immunomodulatory medication used to treat multiple sclerosis (MS). Like sphingosine, fingolimod undergoes phosphorylation to an active metabolite, fingolimod phosphate (FTY720-P), which is structurally analogous to sphingosine-1-phosphate (S1P) (David et al. 2012). FTY720-P functions as an agonist for four of the five isoform receptors of S1P, i.e., S1PR1, S1PR3, S1PR4, and S1PR5 (Don et al. 2007; Hogenauer et al. 2008; Zemann et al. 2006). Yet despite its immediate agonistic action, FTY720-P promotes endocytosis and degradation of the S1P receptors, functioning effectively as an antagonist. FTY720 can distribute across the blood–brain barrier, access the central nervous system (CNS), and localize to brain white matter, where it can alter neuroinflammatory responses of auto-reactive T cells and glial cells (Foster et al. 2007). In the rodent model of MS, experimental autoimmune encephalomyelitis (EAE), FTY720 ameliorated neurological symptoms (Fujino et al. 2003) in a manner that depended on astrocyte S1P1 receptor modulation (Choi et al. 2011). The preclinical data with EAE led to the successful human clinical trials of fingolimod to reduce relapses in MS; and fingolimod is now being explored in other neuroinflammatory and/or neurodegenerative disorders (O'Sullivan and Dev, 2017).

Fingolimod is thought to reduce relapse rates in MS primarily by altering lymphocyte trafficking, that is, by inhibiting egress of lymphocytes from the lymph nodes, preventing lymphocyte migration to the CNS, and thus reducing CNS inflammation. But clinical trials also revealed a potentially beneficial effect of fingolimod on brain volume; study patients on drug had reduced annual rates of brain volume loss (BVL) by about one-third relative to placebo (Calabresi et al. 2014; Kappos et al. 2010). This implies that fingolimod can be neuroprotective in CNS inflammatory disease states. Indeed, for over a decade, emerging lines of research have indicated that fingolimod may act directly on neuroepithelial-derived cell types through S1P receptor-mediated cell signaling events. Fingolimod has been demonstrated to provide neuroprotection against excitotoxic neuronal cell death in vitro and appears to do so via direct action on neuronal S1P1 receptors, independent of astrocytes, and using intracellular pathways that support the survival of neurons (Di Menna et al. 2013). In rodent neuronal cell cultures, fingolimod elicited a neuronal gene expression response that modulated the morphology of actin-rich neuronal growth cones and stimulated neurite growth in vitro (Anastasiadou and Knoll, 2016). There is some evidence in favor of neurotrophic activity (Groves et al. 2013) and reduction of amyloid-β accumulation in Alzheimer’s disease models (Asle-Rousta et al. 2013; Hemmati et al. 2013). But, there remain questions as to the mechanism and scope of fingolimod’s neuroprotective potential during chronic CNS inflammatory diseases. Moreover, there is little information on whether fingolimod can protect neurons in the neuroinflammatory and neurodegenerative microenvironment found in chronic HIV-1 infection of the aging brain and HIV-associated neurocognitive disorders (HAND) (Canizares et al. 2014).

The objective of this study is to identify potential neuroprotective pathways of fingolimod action by examining neuronal gene expression in an inflammatory microenvironment that mimics chronic HIV infection of the CNS. Our previous studies showed that when human neuroepithelial progenitor cells (NEP) are allowed to differentiate in vitro into a mixed population of astrocytes and neurons in the presence of HIV, neurons “fail to thrive” (Geffin et al. 2013; Martinez et al. 2012; McCarthy et al. 2006). There are lower total neurite lengths per cell and moderately decreased amounts of the neurofilament light protein (NF-L) in HIV-exposed neurons. Moreover, these effects occurred even without apparent evidence of productive viral infection (McCarthy et al. 2006). While our previous studies used a mixed glial and neuronal cell population differentiated from human fetal NEP (Geffin et al. 2013; Martinez et al. 2012; McCarthy et al. 2006), for this study, we have adapted a human neural progenitor cell line, hNP1, to emphasize neuronal-specific responses to HIV exposure and fingolimod treatment. The human neural progenitor cell line hNP1 was derived from the NIH-registered human embryonic stem cell (hESC) line WA09 and established as a neural progenitor line that can be differentiated into an enriched population of human post-mitotic neurons. The neurons have functional properties such as ion channels and ionotropic receptors (Dhara et al. 2008; Guo et al. 2013; Young et al. 2011). The hNP1 cells were exposed to HIV_SF2 MC_ or Mock-infected culture supernatants during differentiation conditions that produced a population of neuronal lineage cells enriched for post-mitotic neurons and lacking astroglia (Geffin et al. 2017). This directly exposed the neurons to viral proteins and associated inflammatory factors (Martinez et al. 2012), a microenvironment that can cause neuronal toxicity and even neuronal cell death (Hesselgesser et al. 1998; Maragos et al. 2003; van Marle et al. 2004). Fingolimod phosphate (FTY720-P) was added to the differentiating hNP1 cultures with or without HIV exposure to assess the effects of HIV and fingolimod exposure separately and in conjunction. Because the hNP1-derived neuronal cultures lack a glial fibrillary acidic protein (GFAP)-expressing astrocyte subpopulation, the observed fingolimod effects and differential gene expression profiles represent neuronal-specific responses. This study provides evidence that fingolimod can have downstream neuroprotective benefit through differential gene expression affecting cell signaling, neuronal glucose metabolism, and neuroinflammation.

## Materials and methods

### Culture and maintenance of hNP1 cells

#### Proliferation

hNP1 cells were obtained from Aruna Biomedical (Athens, GA). Undifferentiated hNP1 cells were proliferated in monolayer cultures at a density of 4.0 × 10^4^ cells/cm^2^ in “progenitor medium” based on AB2™ Basal Neural Medium supplemented with 2% AB2 supplement (Aruna Biomedical, Athens, GA), as described previously (Geffin et al. 2017). Cultures were fed every 2 days and passaged when 100% confluency was reached or approximately once per week.

#### Differentiation

For differentiation into neurons, hNP1 cells were seeded onto poly-d,l-ornithine plus fibronectin-coated (PON-FN) coated tissue culture wells at 8.0 × 10^3^ cells/cm^2^. They were cultured in progenitor medium for 2 days, then switched to “differentiation medium” dAB2, modified from AB2 medium by removal of bFGF (Aruna Biomedical, Athens, GA). Cultures were continued in differentiation medium for up to 12 days. Real-time reverse transcription polymerase chain reaction (RT-PCR) confirmed neuronal-specific messenger RNA (mRNA) and immunoblotting confirmed the presence of nestin and post-mitotic neurofilament proteins (Geffin et al. 2017). Neither mRNA nor protein for the major astrocyte intermediate filament protein, GFAP, was detected in the lysates from differentiating hNP1 cultures at any time point.

### Differentiation and culture treatment

Twenty-four hours after the hNP1 cultures were changed to differentiation medium, which was designated experimental day 0, the differentiating cultures were sorted into two groups for incubation with added fingolimod phosphate (FTY720-P) or no added FTY720-P. Each group was further divided into triplicate samples for three culture treatments: HIV-exposed, Mock-exposed, or untreated (Supplementary Fig. S1). Thus, each culture condition was tested in triplicate with or without added FTY720-P. For all culture treatments, with and without FTY720-P, culture media with additives were fully replenished every 3 days.

#### “HIV-exposed”

HIV-1-containing supernatants were obtained from human peripheral blood mononuclear cells (PBMC) that were stimulated with mitogen phytohemagglutinin (PHA, Sigma-Aldrich, St. Louis, MO) and recombinant human interleukin-2 (IL-2, Roche Diagnostics, Indianapolis, IN), then infected with HIV-1 as described previously (McCarthy et al. 1998). When viral concentration in the infected PBMC supernatants was at least 100 ng/ml, the supernatants were harvested, centrifuged at 400×*g* for 10 min to eliminate cellular debris, aliquoted, and frozen at −140 °C until needed. Differentiating hNP1 cultures were exposed to HIV by aliquoting human PBMC-derived stock virus into differentiation culture medium at 20 ng p24 per 7.5 × 10^4^ hNP1 cells. The HIV-1 strain used throughout this study was obtained through the NIH AIDS Reagent Program, Division of AIDS, NIAID, NIH: HIV-1_SF2 MC_, a dual tropic strain, from Dr. Jay Levy. This virus strain recognizes both CXCR4 and CCR5 co-receptors for HIV binding (Trkola et al. 1998).

#### “Mock-exposed” (control)

Mock-exposed hNP1 cultures were used to control for the effects of nonviral inflammatory factors present in mitogen-stimulated PBMC supernatants (Martinez et al. 2012). Mock-infected PBMC supernatants were derived from PBMC stimulated with PHA and IL-2, prepared at the same time and using the same PBMC donor cells that were used to culture the HIV-1_SF2 MC_ stock virus, but never infected with HIV. Then, aliquots of Mock-infected PBMC supernatant, equal in volume to the aliquots used for HIV-1_SF2 MC_-infected PBMC supernatants, were added to parallel differentiating hNP1 cultures.

“Untreated” hNP1 cultures contained only differentiation medium with added aliquots of PBMC growth medium (RPMI + 20% (*v*/*v*) fetal bovine serum) in the same volume as Mock-infected or HIV-infected PBMC supernatants.

### Fingolimod treatment

Fingolimod phosphate (FTY720-P) was received as lyophilized powder from Dr. Paul Smith, Novartis Institutes for Biomedical Research, Basel, Switzerland. Powder was initially dissolved in acidified DMSO to a final concentration of 8 mM. This was then diluted to stock solutions of 1 mM and again to 10 and 1 μM in sterile phosphate-buffered saline (PBS). Dose–response curves were performed to assess cell viability. A final concentration of 10 nM FTY720-P was optimal and was used for the experiments described herein (see “Results”). In experimental cultures, FTY720-P was diluted 1:100 from the stock 1 μM solution into culture medium and replenished daily. In controls not cultured with FTY720-P, the solvent for FTY720-P (acidified DMSO) was diluted 1:8000 in PBS, then 1:100 into culture medium, and also replenished daily. For each culture treatment (untreated, Mock-exposed, and HIV-exposed), separate triplicate cultures were treated with 10 nM FTY720-P or the control diluted solvent solution added to differentiation medium.

After 12 days of incubation, replicate monolayer cultures were harvested for either quantitative immunoblotting or RNA extraction.

### Immunoblotting

The total protein content of whole cell lysates from cell cultures was determined by BCA protein assay (Pierce, Rockford, IL) and between 5 and 10 μg total protein was loaded onto 10% polyacrylamide gels. Quantitative immunoblotting was performed basically as described (McCarthy et al. 2006) with chemiluminescent signal detection to assess amyloid precursor protein (APP) expression. APP was detected with a 1:1000 dilution of rabbit polyclonal antibody (Cell Signaling Technology, Danvers, MA) and a secondary sheep anti rabbit IgG (GE Healthcare, UK) diluted to 1:2000. β-actin was used as an internal control for potential loading errors in each lane; it was detected with an anti-β-actin mouse monoclonal antibody (Sigma, St. Louis, MO). Immunoblots were developed with SuperSignalWest Femto Maximum Sensitivity Substrate (Thermo Scientific, Rockford, IL). Signals were viewed and quantitated using a Bio-Rad Chemidoc XRS+ Molecular Imager; the APP signal density was normalized to β-actin. Quantification of APP and β-actin signals used the average of signals from three independent immunoblot experiments.

### RNA preparation and gene expression microarray generation

hNP1 cultures were incubated in differentiation medium and in the specified culture treatment conditions for 12 days, with replenishment of culture medium and additives at day 11. At day 12, the incubations were terminated and cultures were lysed for preparation of RNA for gene expression determinations. Total RNA was extracted from differentiating hNP1 cells using the Illustra RNAspin Mini kit from GE Healthcare (Pittsburgh, PA). RNA concentrations, 260/280, and 260/230 absorbance ratios were determined using a Nanodrop from Thermo Scientific (Wilmington, DE). Further RNA analyses and microarray data generation were performed by the technical staff of the Oncogenomics Core Laboratory, Sylvester Comprehensive Cancer Center (SCCC), Miller School of Medicine, and the University of Miami.

Total RNA was quantified with a Nanodrop 8000 Spectrophotometer (Thermo Scientific, Wilmington), and its quality was examined with a Bioanalyzer 2100 using the RNA 6000 Nano kit (Agilent, Santa Clara, CA). Biotinylated cRNA was prepared using the Illumina TotalPrep RNA Amplification kit (Ambion, Inc., Austin, TX) according to the manufacturer’s instructions starting with 400 ng total RNA. Successful cRNA generation was checked using the Bioanalyzer 2100. Samples were added to the Beadchip after randomization using the randomized block design to reduce batch effects. Hybridization to the Sentrix Human-HT12 Expression BeadChip (Illumina, Inc., San Diego, CA), washing, and scanning were performed according to the Illumina BeadStation 500 manual (revision C). The resulting microarray data was analyzed using Illumina GenomeStudio software.

### Gene expression microarray analysis

Raw expression data from microarray Human HT-12_v4_0_R1 Illumina platform was obtained for day 12 and was loaded on Bioconductor/R software and a probe level analysis was performed on them. The raw intensity data from the microarray was normalized using the Quantile normalization method, log (base 2) transformed, and scaled the median to 0 of all samples. An unpaired Student’s *t* test for two class comparisons was used to identify the significantly differentially expressed genes between the tested conditions. Two-sided Student’s *t* test was performed with confidence level = 0.95 with two variances being treated as unequal. Multiple testing corrections were not performed on the nominal *p* values. Probes for different genes were considered significantly differentially expressed for those with absolute fold change value (|FC|) ≥ 1.5 and nominal *p* value ≤ 0.05. Functional enrichment analysis was performed on significant genes using the MetaCore module on GeneGO web portal program from Thomson Reuters™ (NY).

### Gene expression validation by quantitative RT-PCR

hNP1 monolayers were incubated in differentiation medium for 12 days, then washed once with PBS, and lysed using the Illustra RNAspin Mini kit from GE Healthcare (Pittsburgh, PA) for RNA extraction. RNA concentrations, 260/280, and 260/230 absorbance ratios were determined using a Nanodrop from Thermo Scientific (Wilmington, DE). Samples were diluted to 10 ng/μl in nuclease-free water. One-step RT-PCR reactions were carried out using a Bio-Rad iTaq Universal SYBR Green 1-step kit from Bio-Rad with 50 ng of RNA per 50 μl reaction and 400 nM of all oligonucleotide primers except for those specific to Adaptor Related Protein Complex 2 Beta 1 Subunit component (AP2B1). Primers were designed using the NCBI primer-BLAST tool (http://www.ncbi.nlm.nih.gov/tools/primer-blast/) with sequences found at the NCBI Refseq website (https://www.ncbi.nlm.nih.gov/refseq/). Primers were designed to anneal to mRNA exons at the 5′ or 3′ side of the junction to avoid amplification of DNA sequences instead of RNA sequences. All primers were synthesized by Life Technologies (Carlsbad, CA). The qRT-PCR samples were run in a Stratagene Mx3005P thermocycler (Agilent Technologies, Santa Clara, CA). The following amplification conditions were used for all genes except AP2B1: 51 °C for 20 min for reverse transcription, an initial denaturing step at 95 °C for 10 min, and then 40 cycles of 95 °C for 10 s and 57 °C for 30 s for annealing and elongation. Melting curves were performed with an initial denaturing step at 95 °C for 1 min and annealing step at 55 °C for 1 min, then 95 °C for 30 s. RT-PCR conditions for amplification of the AP2B1 gene were slightly modified: the concentration of the reverse oligonucleotide primer was 600 nM. The amplification conditions were changed to 51 °C for 20 min for reverse transcription, an initial denaturing step at 95 °C for 10 min, and then 40 cycles of 95 °C for 15 s and 60 °C for 30 s for annealing and elongation. Data was analyzed using the MxPro software (Agilent Technologies, Santa Clara, CA).

Target gene expression was normalized to the housekeeping control gene glyceraldehyde-3-phosphate dehydrogenase (GAPDH) amplified from the same sample. Gene expression was calculated using the ddCT method (Vandesompele et al. 2002). For each culture condition, three independent experimental replicates, i.e., three independent cultures, were used to determine the expression of a gene by RT-PCR. First, triplicate samples from a single experimental replicate were assayed by one-cycle RT-PCR assay, and the mean of the normalized gene expression for that experimental replicate was calculated from the three sample replicates. Fold change in gene expression (FTY720-P versus no FTY720-P) or (Mock versus untreated) or (HIV versus untreated) or (HIV versus Mock) was calculated as a direct ratio of expression values for each individual experimental replicate.

Representative genes chosen for validation by quantitative RT-PCR were those with significant changes in gene expression when comparing added FTY720-P versus no added FTY720-P or when comparing culture treatments (e.g., Mock-exposed versus untreated) with or without added FTY720-P. After validation of the oligonucleotide primers designed for each representative gene, quantitative RT-PCR was performed using the same cellular RNA specimens that were used in the microarray analyses.

Oligonucleotide primers used for this study had the following sequences: annexin 1 (ANXA1) forward 5′GTGTGGCTTCCTTTAAAATC3′ and reverse 5′CATTTTCAATAAACCAGGCC3′; B2M forward 5′AGATAGTTAAGTGGGATCG3′ and reverse 5′AAAGTGTAAGTGTATAAGCAT3′; APP forward 5′CAAAACCTGCATTGATACCA3' and reverse 5′CATCACTTACAAACTCACCA3′; STAT1 forward 5′CAGTAAAGTCAGAAATGTG3′ and reverse 5′TTCATCTTGTAAATCTTCCA3′; TAF15 forward 5′CAGCAAAACATGGAATCATC3′ and reverse 5′CATATGAGCCTTGATGTTGA3′; RBM39 forward 5′AGCATCAAATTAAGACGACG3′ and reverse 5′CTTGCATCTCTTTCCTCAG3′; ID2 forward 5′CACCCTCAACACGGATATC3′ and reverse 5′TTCAGCACTTAAAAGATTCC3′; and AP2B1 forward 5′GGAAAACAGCAGCAGTCT3′ and reverse 5′ATTTCAGATAATGCCGCTACG3′.

## Results

### Effect of Mock or HIV exposure on differential gene expression by hNP1 cells

Our previously published study (Geffin et al. 2017) using the hNP1 cell line showed that, after 12 days in differentiation media, these cells express post-mitotic neurofilament antigens, Hu neuronal nuclear antigen, and β-III-tubulin neuronal microtubule antigen, along with some residual intermediate filament nestin, a marker for neural progenitor cells. However, the intermediate filament GFAP, a marker for astrocytes, was not detected. This indicates that these differentiated hNP1 cultures are primarily post-mitotic neurons, with a subpopulation of residual neuronal progenitor cells expressing nestin.

To assess the impact of Mock or HIV culture treatment on the hNP1 cells, differential gene expression was calculated as a FC (see “Materials and methods”), representing the ratio of a gene’s expression in Mock-exposed versus untreated or HIV-exposed versus untreated hNP1 cultures. Genes with FC having nominal *p* value ≤ 0.05 and |FC| value ≥ 1.5 were identified and then categorized for function.

**Fig. 1:**
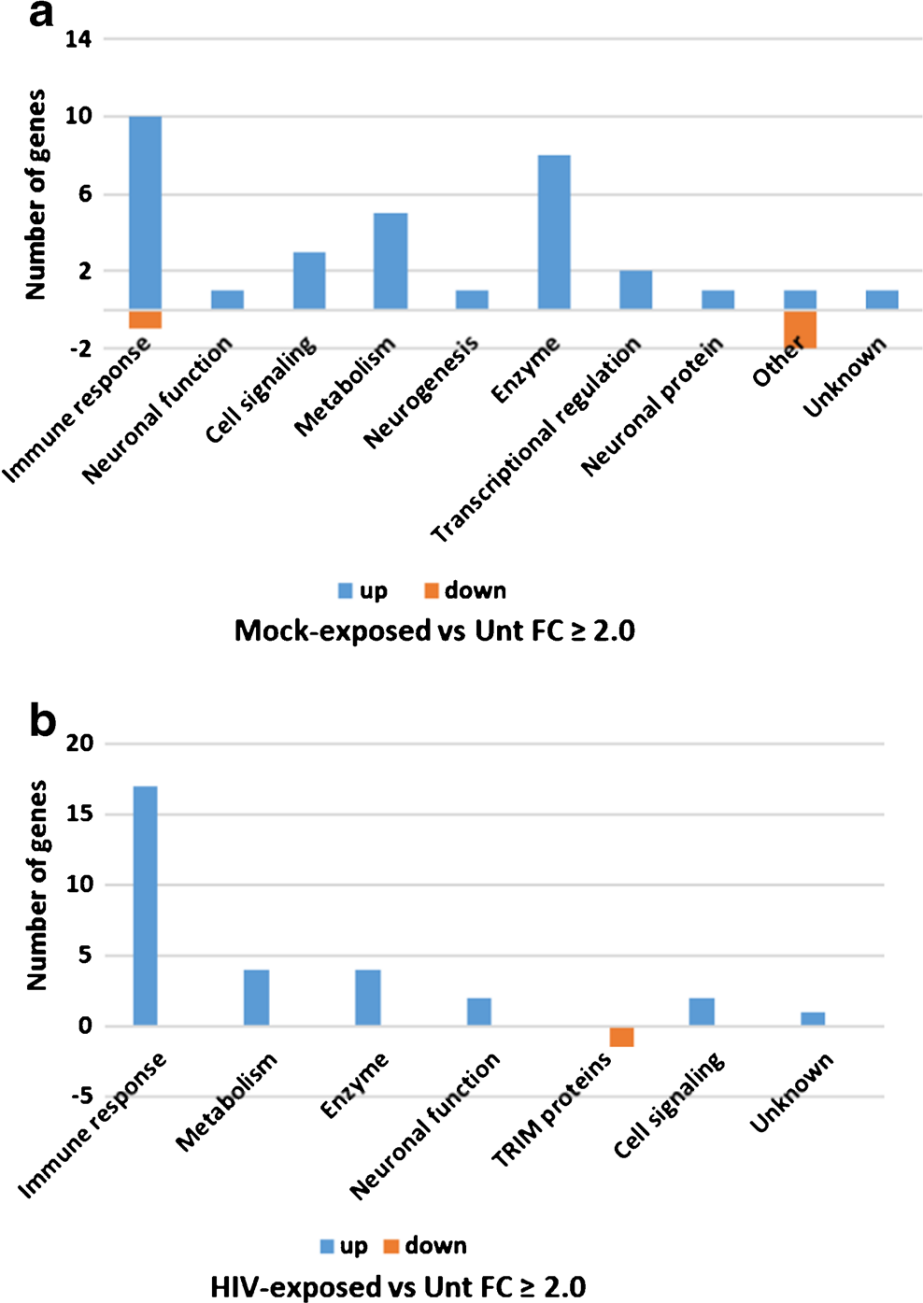
Function profiles of differentially expressed (DE) genes determined from comparisons of **a** Mock-exposed versus untreated cultures and **b** HIV-exposed versus untreated cultures, all without FTY720-P. DE genes with |FC| of ≥ 2.0 and *p* value (nominal) ≤ 0.05 were plotted according to their known biological function

Gene expression microarray revealed that Mock-exposed compared to untreated cultures differentially regulated a total of 167 genes, 112 upregulated and 55 downregulated (Supplementary Table S1). Of these, 33 were upregulated and 3 were downregulated with an |FC| value of 2.0 or greater (Fig. 1a and Supplementary Table S2). Of the 33 genes upregulated by Mock exposure, 10 (10/33, 30%) were related to immune response, mainly interferon-related genes. Other genes included those coding for enzymes and proteins related to cellular metabolism (Fig. 1a).

HIV-exposed compared to untreated cultures differentially regulated 103 genes with an |FC| value of 1.5 or more, 70 upregulated and 33 downregulated (Supplementary Table S1). Of these, 30 were upregulated and 2 were downregulated with an |FC| value of 2.0 or more (Fig. 1b and Supplementary Table S2). A large number of the genes differentially regulated with an |FC| of 2.0 or more included those associated with immune response (17/30 upregulated genes, 56.7%). Immune response genes included HLA genes, interferon responsive genes, and other genes associated with antigen presentation. Other genes affected by HIV exposure were genes coding for enzymes and general cellular metabolism (Fig. 1b). Approximately 2/3 of the genes differentially regulated by HIV as compared to untreated cultures are also differentially regulated in at least one of several similar analyses of primary human neural cultures or brain (Supplementary Table S3). Gene expression changes comparable to that of HIV-exposed hNP1 cells are found in mixed glial and neuronal cultures differentiated from human neuroepithelial cells in vitro (Geffin et al. 2013) or in brain tissues from HIV-infected humans or Simian Immunodeficiency virus (SIV)-infected macaques (Borjabad et al. 2011; Geffin et al. 2013; Gelman et al. 2012; Masliah et al. 2004; Roberts et al. 2003).

Fold change in gene expression was also determined for HIV-exposed versus Mock-exposed cultures. This approach discerns the specific effect of HIV exposure, separate from the potential effects that factors present in mitogen-activated, Mock-infected PBMC supernatants might have on the differentiating hNP1 cultures. HIV exposure compared to Mock exposure differentially upregulated nine genes with an |FC| value of ≥ 1.5, all of them related to immune response (Supplementary Table S1). Three genes, TAP1, HLA-B, and PSMB9, were differentially expressed with an |FC| value of more than 2.0.

A number of genes (*n* = 20) that were differentially expressed with |FC| greater than 2.0 were common to Mock-exposed versus untreated and HIV-exposed versus untreated cultures (Supplementary Table S2). Approximately half of these common genes are implicated in immune responses. These included B2M, IFITM1, IFITM2, IFITM3, IRF9, ISG15, STAT1, TAP1, and ZC3HAV1. The remaining genes differentially expressed in common coded for enzymes or proteins with metabolic cellular functions. Two genes coded for proteins with cell signaling functions, ELMOD1 and FAM167A (Supplementary Table S2).

### FTY720-P treatment of differentiating hNP1 cells

**Fig. 2:**
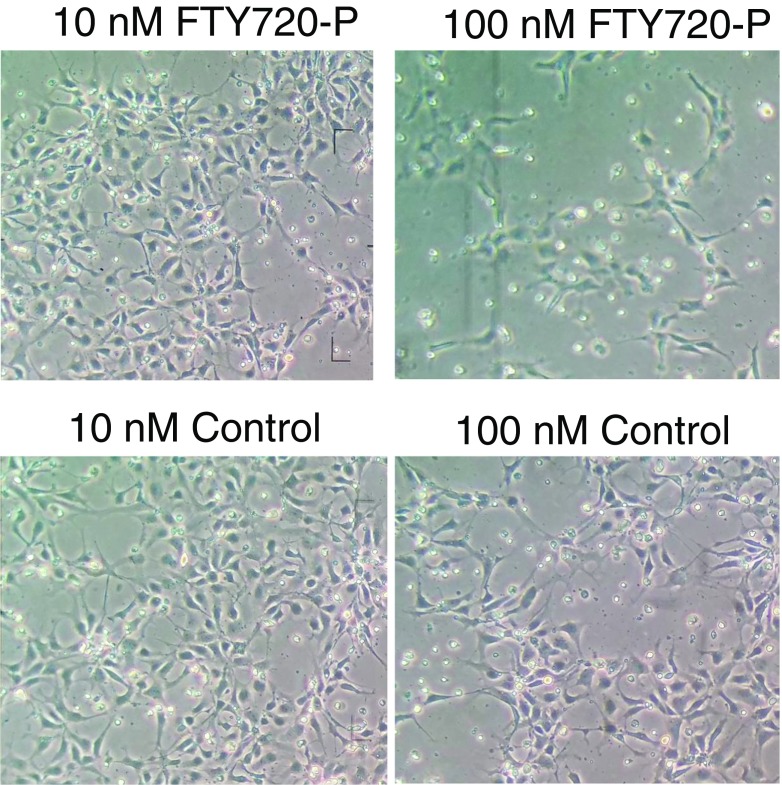
Phase contrast microscope images of hNP1 cell cultures treated with FTY720-P. hNP1 cultures in differentiation medium were exposed to 10 and 100 nM of FTY720-P or equivalent volumes of FTY720-P dilution medium (control) added to the cultures for 1 day. Cultures were then photographed under phase contrast (×400). Lower cell densities were observed in cells treated with 100 nM FTY720-P, compared to the corresponding control culture

Fingolimod at micromolar concentration has been reported to have some toxic effects on adherent human astrocyte cultures (Wu et al. 2013) and cortical neurons from rat embryos (Cipriani et al. 2015). Accordingly, we assayed nanomolar range concentrations of FTY720-P for apparent toxicity and loss of cell viability in differentiating hNP1 cultures (Fig. 2). After 1 day of culture, at a concentration of 100 nM FTY720-P, cell density was approximately one-third that of the corresponding control culture. Moreover, cell density in the control culture for 100 nM FTY720-P, which contained the equivalent dilution of FTY720-P solvent (acidified DMSO), was approximately one-half that of the cultures treated with 10 nM FTY720-P or corresponding control. Cell viability was similar in 10 nM FTY720-P-treated and corresponding control (Fig. 2). DMSO at the concentrations used in the 10 nM FTY-720P solvent was not directly toxic to the cells. Thus, 10 nM FTY720-P was subsequently used to assay fingolimod effects on hNP1 cells cultured in differentiation medium for 12 days. FTY720-P activity in the cells was detected as increased phosphorylation of extracellular-signal regulated kinase (ERK) protein (Deogracias et al. 2012) (data not shown). Akt and phosphorylated Akt were also detected in FTY720-P-treated cultures (data not shown). FTY720-P treatment had no significant effect on the expression of mRNA for neuroepithelial markers nestin, β-III-tubulin, neurofilament-L, or MAP2, determined by RT-PCR of mRNA harvested at 12 days of culture (data not shown). The mRNA for GFAP was not detected in either undifferentiated or differentiated cells treated with FTY720-P, consistent with our previous experience with these culture conditions (Geffin et al. 2017).

### Effect of FTY720-P treatment on differential gene expression by hNP1 cells

#### Mock exposure versus untreated

**Fig. 3:**
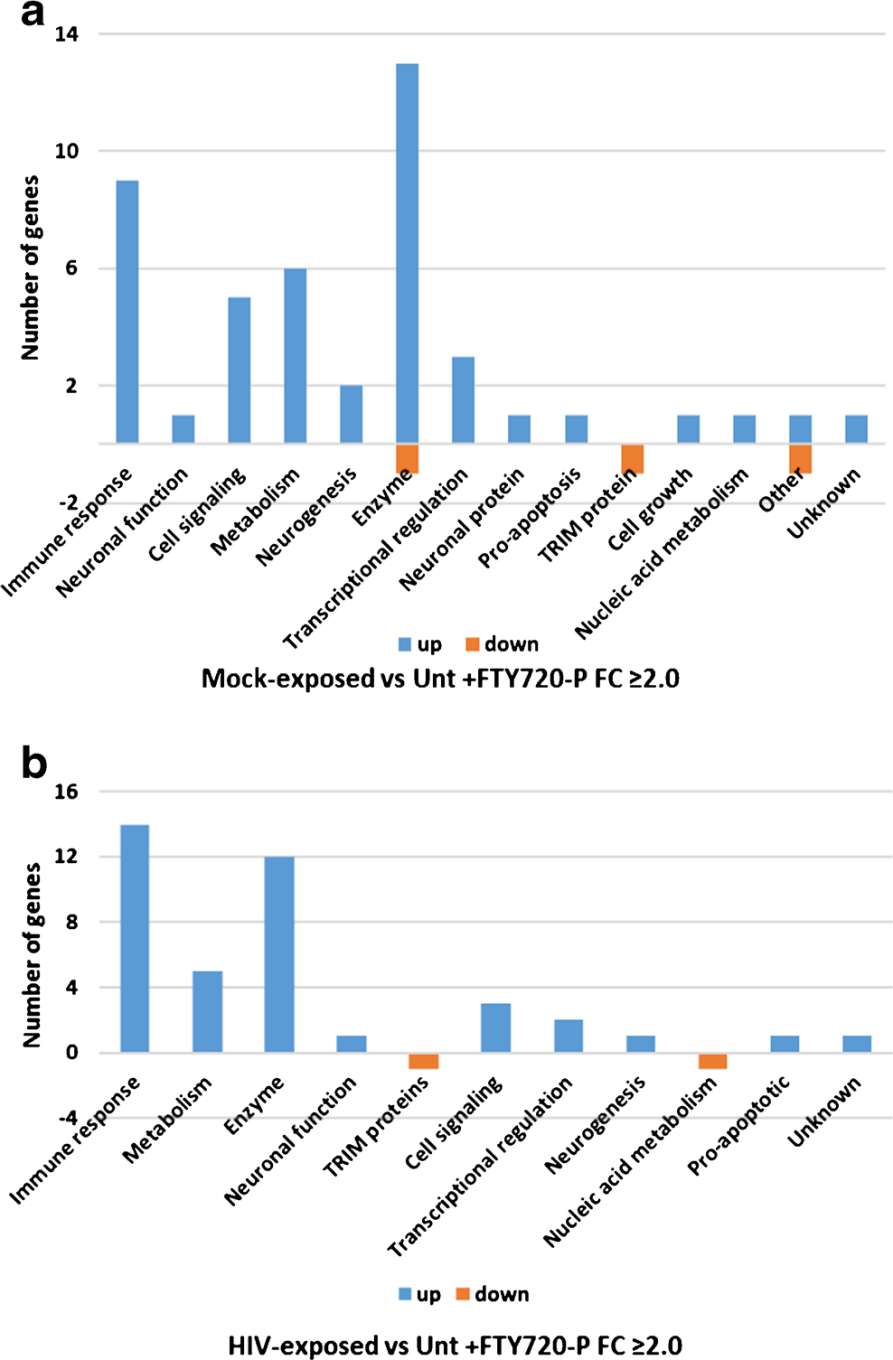
Function profiles of differentially expressed genes determined from comparisons of **a** Mock-exposed versus untreated cultures and **b** HIV-exposed versus untreated cultures, all with FTY720-P present in differentiation medium for the 12-day incubation. DE genes with |FC| ≥ 2.0 and *p* value (nominal) ≤ 0.05 were plotted according to their known biological function

In the presence of FTY720-P, Mock exposure of hNP1 cultures differentially regulated 235 genes with an |FC| of 1.5 or more (Mock-exposed versus untreated) (Supplementary Table S4). Of these, 144 were upregulated and 91 downregulated, while 45 were upregulated and 3 downregulated with an |FC| value of 2.0 or more, a total of 48 genes (Fig. 3a and Supplementary Table S5). Again, prominent among the genes differentially regulated are those coding for enzymes and proteins involved in the immune response (Fig. 3a). Among the S1P receptor genes, two were significantly downregulated, though not to a great extent, S1PR3 (FC = −1.61, nominal *p* = 0.012) and S1PR1 (FC = −1.25, nominal *p* = 0.005). The other S1PR genes were not differentially affected. This modest effect of FTY720-P exposure on S1P receptor gene expression is consistent with the modest effects reported for FTY720-P-treated rodent neurons (Anastasiadou and Knoll, 2016). The 235 differentially regulated genes with an |FC| of 1.5 or more constitute a 40% increase in differentially regulated genes as compared to Mock-exposed cultures without added FTY720-P. Moreover, the percent of differentially regulated genes coding for enzymes or proteins involved in metabolism increased in the presence of FTY720-P in both Mock-exposed and HIV-exposed cultures as compared to untreated cultures, while the percent of those coding for immune-related genes decreased in the presence of FTY720-P.

Of the 48 genes that were differentially regulated with an |FC| of 2.0 or more (Fig. 3a), 29 of these were differentially regulated by Mock exposure (Mock-exposed versus untreated) in the presence or absence of FTY720-P (Supplementary Table S6). These included genes for immune-related responses, enzymes, metabolism, and cell signaling. The remaining 19 genes were uniquely differentially regulated in the presence of FTY720-P at a threshold |FC| of 2.0 or more. However, 17 of those 19 genes were also significantly differentially regulated in the absence of FTY720-P, but the |FC| values (Mock-exposed versus untreated) were just below 2.0. This underscores the significant effect of Mock exposure on gene expression by the differentiating hNP1 cells. In Mock-exposed cultures, fingolimod treatment tended to intensify gene expression but did not change the identities of the most differentially expressed genes.

#### HIV exposure versus untreated

In the presence of FTY720-P, HIV exposure of hNP1 cultures differentially regulated 137 genes with an |FC| of 1.5 or more (HIV-exposed versus untreated) (Supplementary Table S4). Of these, 105 were upregulated and 32 were downregulated, while 40 were upregulated and 2 were downregulated with an |FC| of 2.0 or more, a total of 42 genes (Fig. 3b and Supplementary Table S5). The proteins coded by these genes include immune response genes such as TAP1, B2M, IFITM1, IFITM3, and IRF9, among others, and proteins with enzymatic activity. Among the S1P receptor genes, only S1PR1 was significantly differentially affected, minimally downregulated with FC = −1.21, nominal *p* = 0.048. The other S1PR genes were not differentially affected. The 137 differentially regulated genes with an |FC| of 1.5 or more constitute a 33% increase in differentially regulated genes as compared to HIV-exposed cultures without added FTY720-P.

Of the 42 genes that were differentially regulated with an |FC| of 2.0 or more (Fig. 3b), 25 of these were differentially regulated by HIV exposure (HIV-exposed versus untreated) in the presence or absence of FTY720-P (Supplementary Table S7). Thirteen of these 25 genes (52%) were genes related to immune responses. Seventeen genes were differentially regulated by HIV exposure only in the presence of FTY720-P. Of the 17 genes that were uniquely differentially regulated in the presence of FTY720-P at an |FC| of 2.0 or more, the majority (15/17, 88%) had an |FC| above 1.5 in corresponding HIV-exposed versus untreated cultures not treated with FTY720-P. However, the *p* values for 12 of these 17 genes (12/17, 76%) were not significant in the cultures that had not been treated with FTY720-P. This suggests that FTY720-P treatment altered the profile of the most differentially expressed genes during HIV exposure, adding 12 significantly expressed genes. The 12 additional genes that were significantly expressed in the presence of FTY720-P were identified as BNIP3, ADM, ALDOC, ID3, PLOD2, PFKFB4, P4HA1, ALDOA, LDHA, HK2, MTP18, and PGK1. Interestingly, six of these genes (ALDOC, PFKFB4, ALDOA, LDHA, PGK1, and HK2) code for proteins involved in aerobic and anaerobic glycolysis.

#### HIV versus Mock exposure

Another differential effect of HIV exposure in the presence of FTY720-P was realized by identifying which genes were differentially regulated when comparing HIV exposure to Mock exposure. This analysis identified 14 genes differentially regulated with an |FC| of 1.5 or more (HIV-exposed versus Mock-exposed) (Supplementary Table S4). Of these, 11 genes were upregulated and 3 genes were downregulated. Of the genes that were differentially regulated, four were related to immune response (HLA-A, HLA-B, STAT1, and TAP1) but the rest coded for proteins with various functions, including transcriptional regulation and histone modification (WDR5, ZNF629, and ZNF682), cell morphology, plasma membrane dynamics (CAPZB and PALM), and regulation of cell proliferation and differentiation (OCIAD1 and TAF15), among others. This is in contrast with the genes differentially expressed in the pairwise comparison of HIV versus Mock exposure in the absence of FTY720-P, where all nine upregulated genes coded for proteins with immune functions (Supplementary Table S1).

### Functional enrichment analyses

Functional enrichment analyses were performed to investigate how cellular pathways and processes were affected by HIV exposure and by treatment with fingolimod. For these analyses, the input gene sets were the differentially expressed genes derived from the pairwise comparisons between Mock-exposed versus untreated or HIV-exposed versus untreated cultures in the presence or absence of FTY720-P. Differentially expressed genes were included if |FC| were 1.3 or higher and nominal *p* values were ≤ 0.05.

**Fig. 4:**
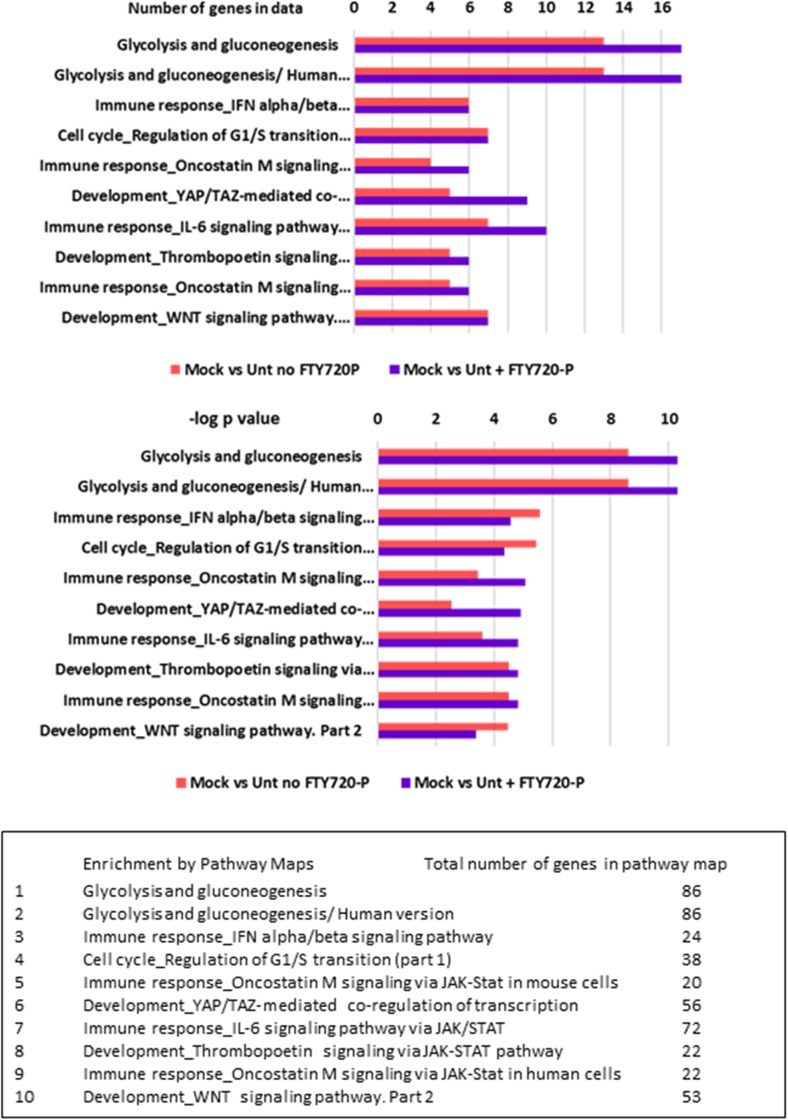
Canonical pathways enriched by Mock exposure in the presence and absence of FTY720-P. The input gene sets were differentially expressed genes determined from the pairwise comparisons of Mock-exposed versus untreated cultures with |FC| ≥ 1.3 and *p* value (nominal) ≤ 0.05, with or without 10 nM FTY720-P treatment. The top 10 canonical pathways are listed by the number of genes enriching the pathways (upper panel) and the −log *p* value of their significance (lower panel). The names of the canonical pathways as well as the total number of genes involved in each pathway are listed in the text box

Of the canonical pathways affected by Mock exposure in the presence or absence of FTY720-P, the two most significant pathways are related to glycolysis and gluconeogenesis (Fig. 4 and Supplementary Table S8). For these pathways, a slightly higher number of genes in data (17 versus 13) and a slightly more significant *p* value (4.98 × 10^−11^ versus 2.5 × 10^−9^) are found in the presence of FTY720-P compared to its absence. The total number of genes involved in these top two canonical pathways is 86, so about 20% of the genes in the pathway are enriched by Mock exposure. Other pathways affected are those involved in immune response signaling (Fig. 4). The number of genes affecting these pathways was about half that of the glycolysis and gluconeogenesis pathways. However, the number of genes enriching three of the affected immune response signaling pathways was 20–30% higher in the presence of fingolimod than in its absence (pathways 5, 7, and 9 in Fig. 4).

**Fig. 5:**
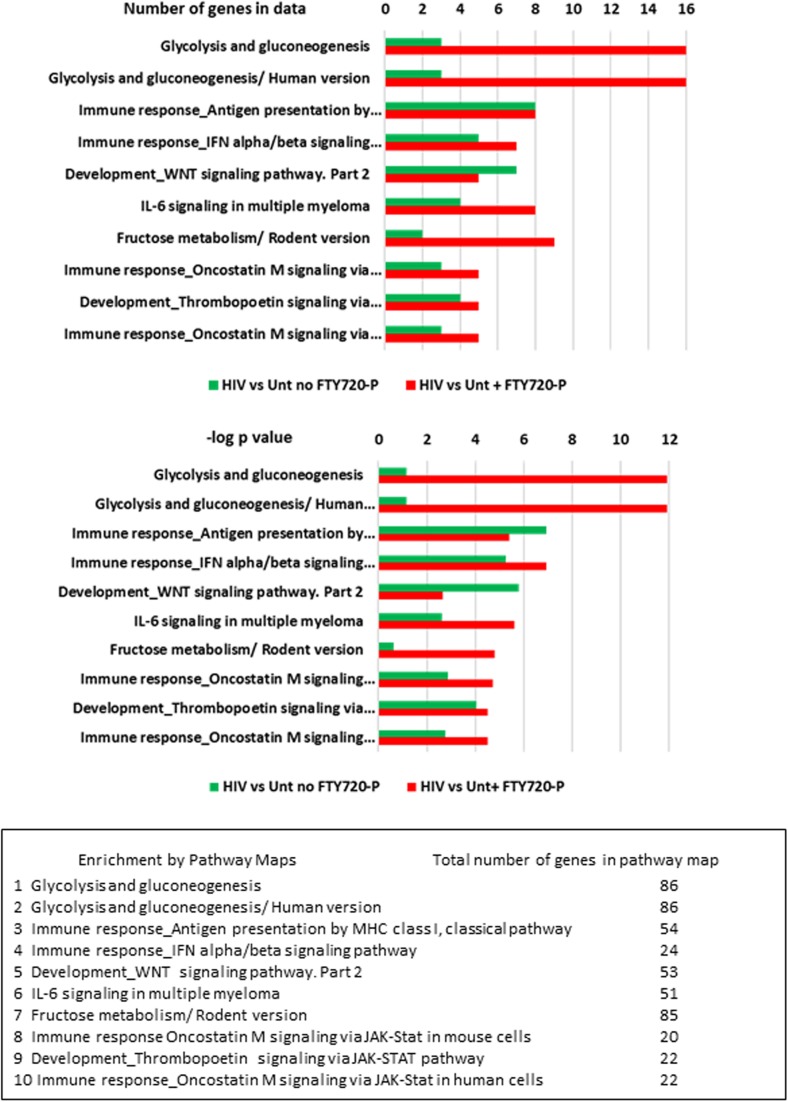
Canonical pathways enriched by HIV exposure in the presence and absence of FTY720-P. The input gene sets were differentially expressed genes determined from the pairwise comparisons of HIV versus untreated cultures with |FC| ≥ 1.3 and *p* values (nominal) ≤ 0.05, with and without 10 nM FTY720-P. The top 10 canonical pathways are listed by the number of genes enriching the pathways (upper panel) and the –log *p* value of their significance (lower panel). The names of the canonical pathways as well as the total number of genes involved in each pathway are listed in the text box

The canonical pathways affected by HIV exposure revealed a more dramatic effect of fingolimod (Fig. 5). As with the Mock exposure, the top two canonical pathways enriched by HIV exposure in the presence of FTY720-P were related to glycolysis and gluconeogenesis (Fig. 5 and Supplementary Table S9), and 16 genes enriched these 2 pathways. However, in the absence of FTY720-P, only three genes enriched the two pathways. The *p* value reflecting the significance of genes enriching these two pathways was also much higher (less significant) in the absence of FTY720-P, 6.94 × 10^−2^ versus 1.2 × 10^−12^ with FTY720-P. In addition to the glycolysis and gluconeogenesis pathways, fructose metabolism (seventh of top 10 pathways) was much more enriched in the presence of FTY720-P (nine genes versus two genes without FTY720-P). Other pathways affected by HIV exposure to a greater extent with FTY720-P treatment included multiple immune response pathways related to antigen presentation and cell signaling (Fig. 5 and Supplementary Table S9).

This functional enrichment analysis ascertained two important sets of functional pathways that are differentially affected by Mock or HIV exposure in the presence of FTY720-P: glycolysis/gluconeogenesis and immune responses. To further specify differentially expressed genes related to glycolysis or immune response functions, the sets of differentially expressed genes from pairwise comparison of culture treatments (Mock-exposed versus untreated, HIV-exposed versus untreated) were examined for genes with these functional relationships. For this analysis, genes with |FC| ≥ 1.5 and nominal *p* value ≤ 0.05 with FTY720-P treatment were included first, then these genes were compared for FC values in corresponding cultures not treated with FTY720-P.

**Fig. 6:**
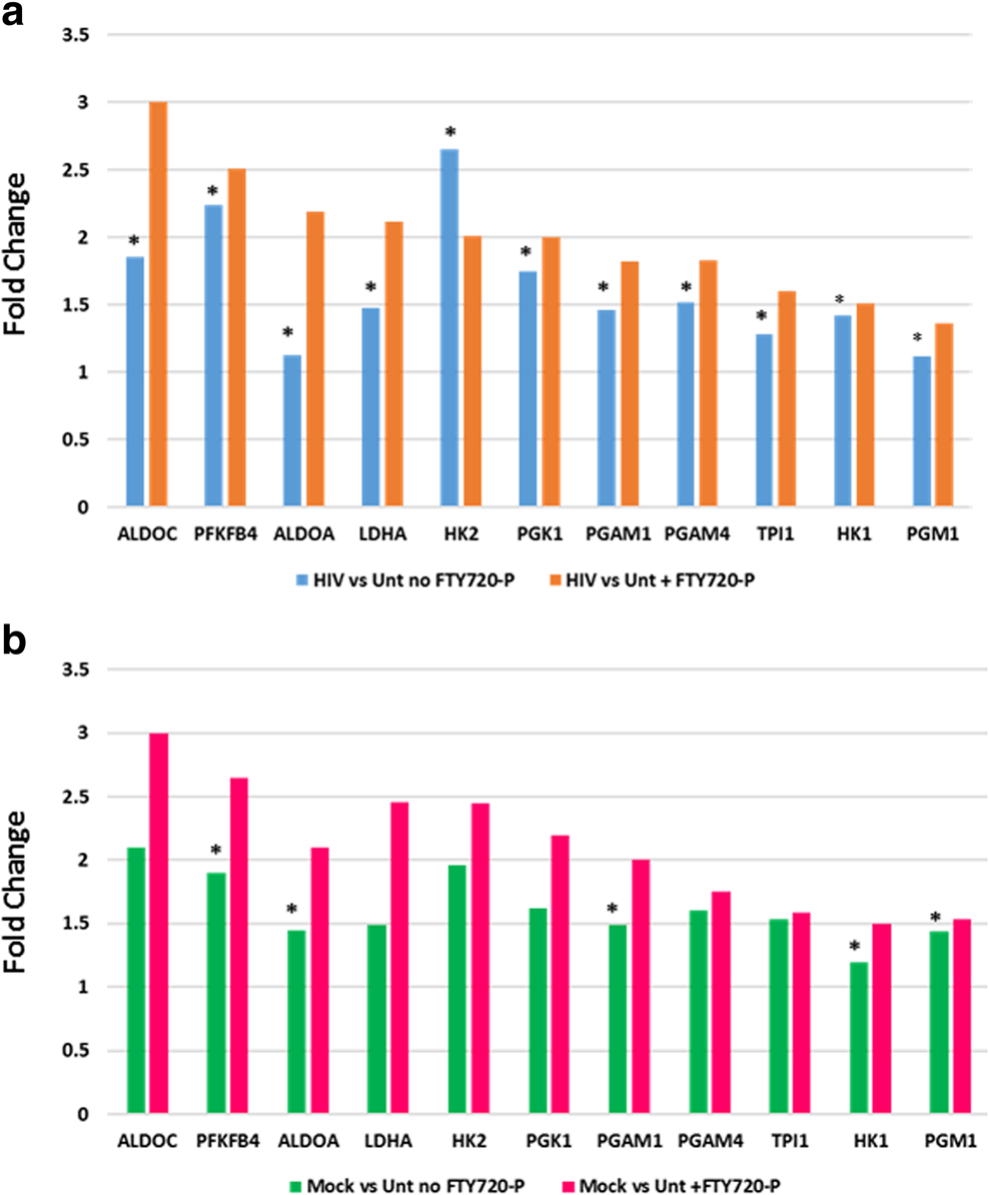
Glycolysis-related genes differentially expressed in hNP1 cultures in the presence or absence of FTY720-P. Genes coding for various glycolysis-related functions were identified from the genes enriching the top 2 canonical pathways in the comparisons of culture treatments shown in Figs. 4 and 5. The FC values for these genes were taken from the pairwise comparisons between **a** HIV-exposed versus untreated cultures and **b** Mock-exposed versus untreated cultures, with and without added FTY720-P. Asterisks (*) denote FC values that were not significant (*p* value (nominal) > 0.05).

Inspection for genes related to glycolytic processes found 11 glycolysis-related genes that were differentially expressed with HIV exposure (HIV-exposed versus untreated) in the presence of FTY720-P (Fig. 6a). Six of these genes (ALDOC, PFKFB4, ALDOA, LDHA, PGK1, and HK2) code for proteins involved in glycolysis and have FC values of 2.0 or higher. An additional five glycolysis-related genes were significantly upregulated by HIV exposure in the presence of FTY720-P but with somewhat lower FC values (Fig. 6a). These included PGAM1 (FC = 1.9), PGAM4 (FC = 1.83), TPI1 (FC = 1.6) HK1 (FC = 1.5), and PGM1 (FC = 1.4). In the absence of FTY720-P, HIV exposure upregulated the expression of 9 of these 11 genes, but nominal *p* values were not significant for any of the 11 genes in the absence of FTY720-P.

The 11 glycolysis-related genes were also upregulated by Mock exposure (Mock-exposed versus untreated) in the presence of FTY720-P (Fig. 6b). While all of them were differentially upregulated with significant *p* values in the presence of FTY720-P, 6 of the 11 were significantly differentially expressed in the absence of FTY720-P. Thus, all the glycolysis-related genes reviewed were differentially upregulated by either Mock or HIV exposure in the presence of FTY720-P. However, in the absence of FTY720-P, for the Mock-exposed versus untreated comparison, only 6 of the 11 genes were significantly differentially expressed. Even more remarkable, for the HIV-exposed versus untreated comparison, none of the 11 genes were significantly differentially expressed. This analysis suggests that fingolimod promotes glycolysis metabolism in differentiating hNP1 cultures, not only when exposed to Mock-infected supernatant but even more so when exposed to HIV-containing supernatant.

**Fig. 7:**
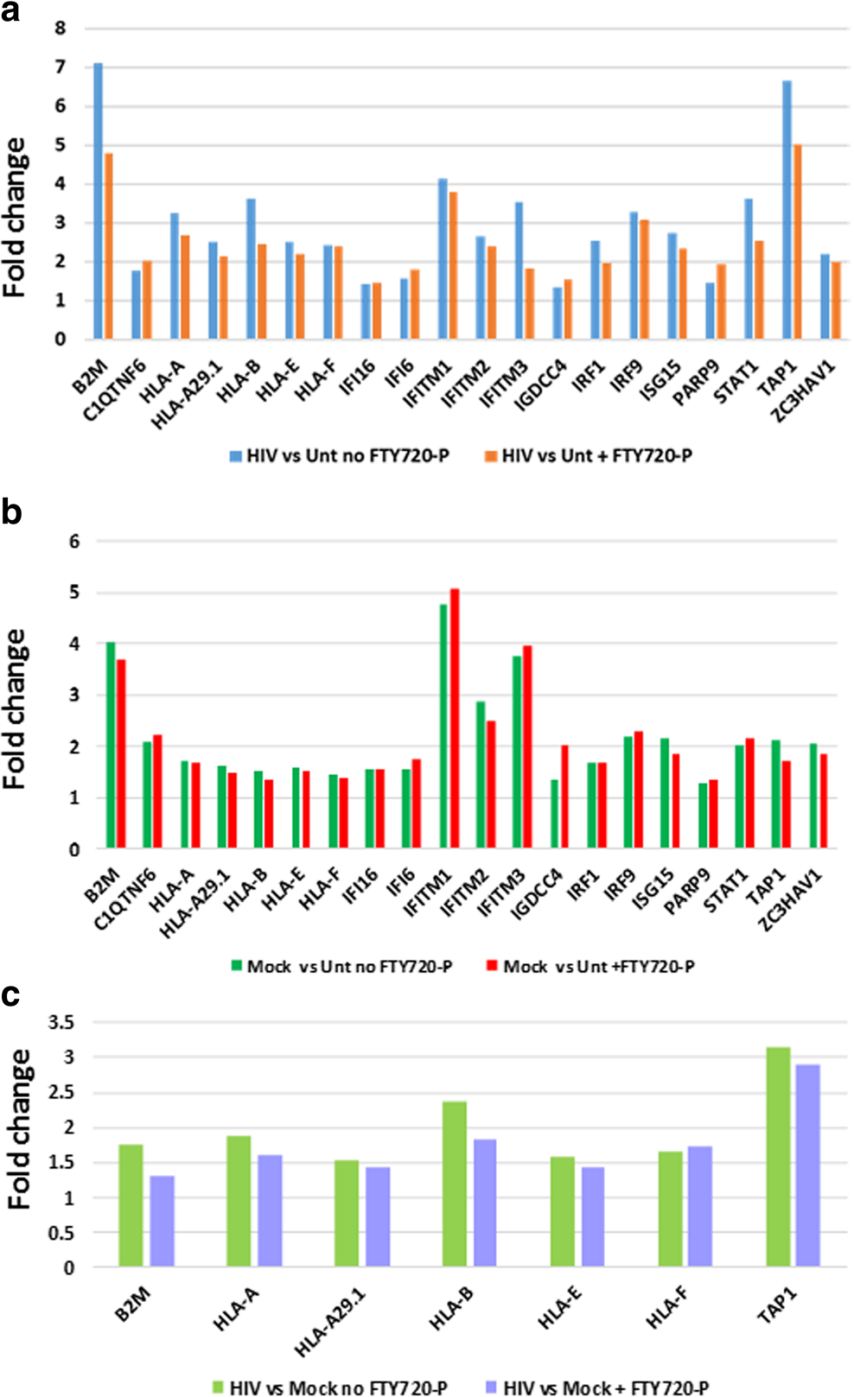
Immune response-related genes differentially expressed in hNP1 cultures in the presence or absence of FTY720-P. Genes coding for proteins with innate immunity and antigen presentation functions were identified from the list of differentially expressed genes with |FC| greater than 1.5 in at least one pairwise comparison, either **a** HIV-exposed versus untreated, **b** Mock-exposed versus untreated, or **c** HIV-exposed versus Mock-exposed cultures, with or without FTY720-P. All the FC values shown have a significant *p* value (nominal) of ≤ 0.05

Inspection for genes related to immune responses found that with either Mock- or HIV exposure, the genes associated with immune response are all differentially upregulated (Fig. 7) when compared to untreated cultures. These include genes related to innate immunity (interferon-related) and antigen-presenting genes. Unlike the genes related to glycolysis (see above), all the FC values obtained for differential immune gene expression have significant nominal *p* values. Interestingly, the expression of these immune-related genes in the pairwise comparison of Mock exposure versus untreated culture is very similar whether FTY720-P is added to the cultures or not (Fig. 7). But in the context of HIV (HIV exposure versus untreated culture), FTY720-P treatment dampens the expression of immune response genes to a variable extent (Fig. 7a), as manifested by FC values that trended lower with FTY720-P treatment (Fig. 7a). This was particularly evident with genes B2M, HLA-B, IFITM3, STAT1, and TAP1, where the FC values were 30% lower with FTY720-P treatment than without. Immune-related genes were also upregulated by Mock exposure (Fig. 7b), generally with lower FC values than found with HIV exposure but with similar FC values with or without FTY720-P. Interestingly, pairwise comparisons of HIV versus Mock exposure revealed that all the seven genes that are differentially expressed are antigen-presenting genes (Fig. 7c), while in the individual comparisons of HIV versus untreated and Mock versus untreated both antigen-presenting and interferon-related genes are upregulated (Fig. 7a, b).

Functional enrichment analyses were also used to examine the direct effect of fingolimod on cellular pathways and processes within a single culture treatment. For this analysis, the input gene sets were the differentially expressed genes derived from a comparison between added FTY720-P versus no added FTY720-P in untreated, Mock-exposed, or HIV-exposed cultures. Differentially expressed genes were included if |FC| were 1.3 or higher and nominal *p* values were ≤ 0.05 (Supplementary Table S10). Only four canonical pathways were found to be significantly affected by fingolimod in all three culture treatments, and the only gene enriching the pathways was follistatin (FST) (Supplementary Table S11). FST was downregulated by FTY720-P treatment in all three culture treatments (Untreated FC = −1.53, Mock FC = −1.31, HIV FC = −1.30) (Supplementary Table S10). Two pathways were related to development, and two pathways were related to signal transduction, including activin A signal regulation. The *p* value for each of the pathways was similar in all culture treatments, indicating that pathways involving follistatin are generally affected by fingolimod. Gene ontology (GO) processes were also queried, and all of the top 10 GO processes affected were related to immune responses for all 3 culture treatments (Supplementary Table S12). The number of genes in data for the top 10 GO processes was 5–10-fold higher in HIV-exposed cultures; however, most of the genes were variants of the HLA-A and HLA-B genes. Other genes affecting the various GO processes in the HIV-exposed cultures were APP, AP2B1, protein phosphatase 3 regulatory subunit (PPP3R1), Lamin B (LMNB), and suppressor of cytokine signaling family (SOCS2), among others. ANXA1 was found to enrich all 10 GO processes for Mock-exposed and untreated cultures. The ID1, ID2, and ID3 genes, which code for transcriptional regulators, were prominently found among the genes in data for untreated cultures affected by fingolimod.

### Expression of APP in lysates of hNP1 cells exposed to HIV and FTY720-P

**Fig. 8:**
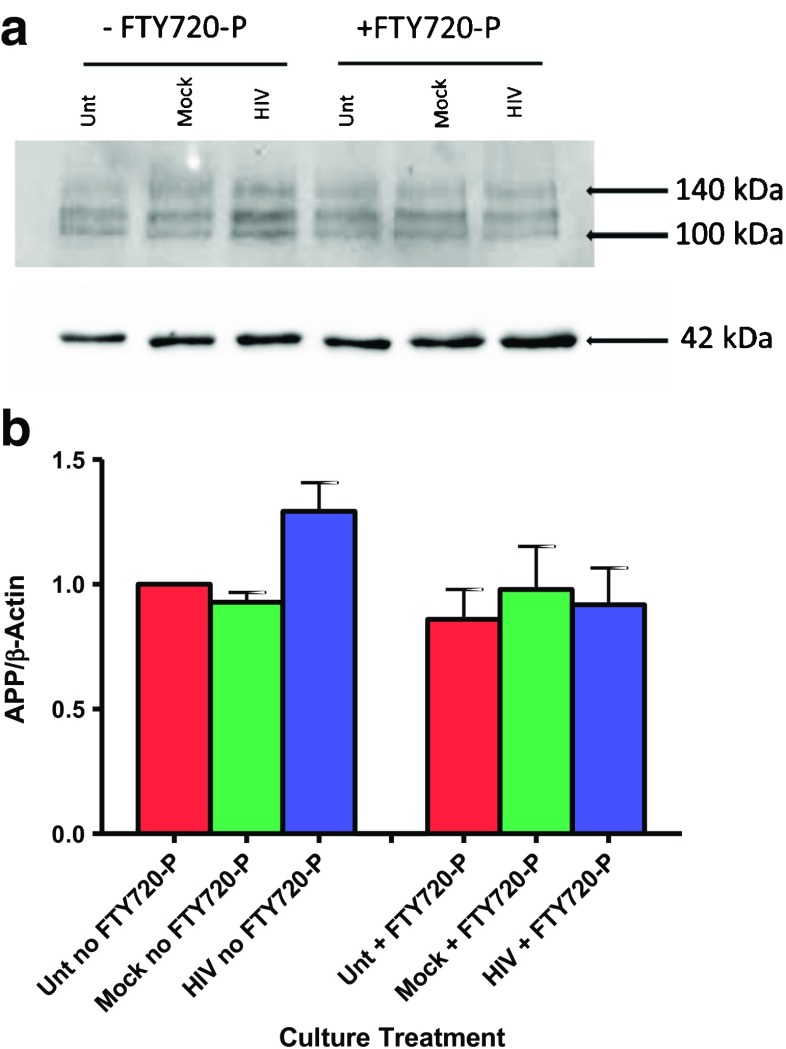
Changes in amyloid precursor protein (APP) protein expression in HIV-exposed hNP1 cells. hNP1 cultures in differentiation medium alone (Unt), Mock-exposed (Mock), or HIV-exposed (HIV), with or without FTY720-P, were lysed after 12 days for quantitative immunoblotting. **a** Representative immunoblot showing the migration of APP as three bands of molecular weight 100 to 140 kDa. Total APP signal was calculated as the sum of signals from the three bands. β-actin, molecular weight 42 kDa, is included as a loading control. **b** Normalized APP signal is the total APP signal divided by β-actin signal in each lane. To compare APP signal across cultures, the normalized APP signal values for each of the culture treatments were divided by the normalized APP signal value for untreated cultures with no FTY720-P. Then, the normalized APP signal value for untreated/no FTY720-P cultures was set to 1. Bar graphs depict the mean ± standard error of normalized APP signal from three independent immunoblots

APP was differentially affected by FTY720-P only in HIV-exposed neurons, where it was downregulated (FC = −1.53, *p* value (nominal) = 0.005, HIV-exposed with FTY720-P versus without FTY720-P). The APP gene was also in data for 7 of the top 10 GO processes (Supplementary Table S12). Given the implications of APP mRNA downregulation for neurodegenerative disease mechanisms, we used immunoblotting to examine APP protein expression in whole cell lysates from hNP1 cells harvested after 12 days in differentiation medium (Fig. 8). Mock exposure did not affect the levels of APP in the hNP1 neurons as compared to untreated cultures, regardless of whether FTY720-P was added or not. However, HIV exposure resulted in a 30% higher normalized APP protein signal as compared to untreated cultures; the increase was then abrogated by FTY720-P treatment.

### Confirmatory RT-PCR of differentially regulated genes

**Fig. 9:**
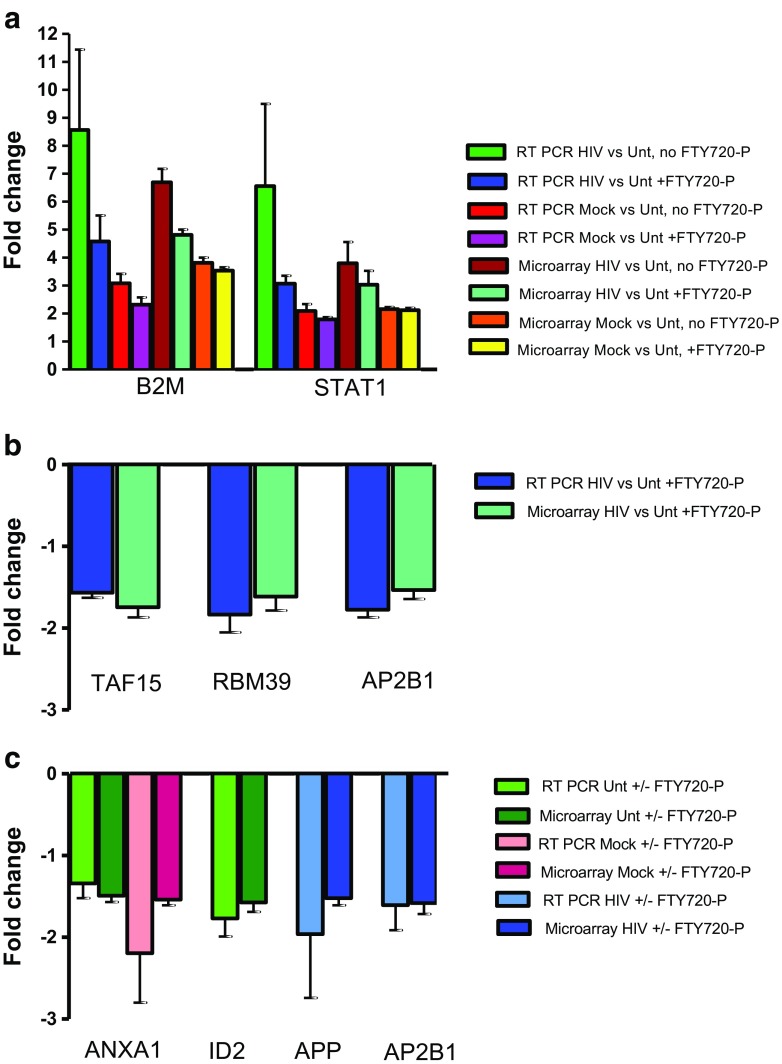
Validation of gene expression microarray FC values by RT PCR. Selected genes had significant FC values for the pairwise comparisons of Mock-exposed versus untreated or HIV-exposed versus untreated or for comparison of added versus no added FTY720-P in a given culture treatment. Selected genes were tested for differential expression as determined by RT-PCR (“Materials and methods”). Lysates used for RT-PCR were the same as those used for the gene expression microarray analysis. FC values for both methods were calculated for each of three individual experimental replicates. Bar graphs depict the mean FC ± standard error from three experimental replicates. Differentially upregulated (**a**) or downregulated (**b**) genes from either Mock-exposed or HIV-exposed versus untreated comparisons are shown. Panel **c** depicts genes that were downregulated in the comparison of FTY720-P versus no FTY720-P within HIV-exposed or Mock-exposed or untreated cultures. For genes with several probes included in the gene array platform, the expression of the probes with highest FC values that had significant nominal *p* values are plotted

RT-PCR was used to independently confirm the differences in gene expression derived from microarray analysis of the hNP1 cultures. Representative genes were selected to validate changes in gene expression as a consequence of FTY720-P addition to the cultures or as a consequence of culture treatment with Mock-infected or HIV-infected PBMC supernatants. The following genes were selected: among the genes that were upregulated by Mock or HIV exposure, immune genes B2M and STAT1; among the genes that were downregulated by HIV exposure, TAF15, RBM39, and the adaptor protein AP2B1; and among the genes that were differentially regulated by fingolimod in the context of each of the culture treatments, ID2 (representing untreated), ANXA1 (representing untreated and Mock-exposed), and APP (representing HIV-exposed). Results obtained represent values obtained by calculating the individual FC values for each culture replicate, both for the microarray and the RT-PCR data. The data presented in Fig. 9 reveal a very close agreement between the FC values obtained by the microarray and the RT-PCR data, which supports the gene expression results obtained in this study.

## Discussion

In this in vitro study, functional genomic analysis was applied to HIV-exposed human neurons and Mock-exposed controls in order to discover potential neuroprotective benefits of fingolimod phosphate (FTY720-P), an analogue of sphingosine-1-phosphate and a current immunomodulatory therapy for multiple sclerosis. In the absence of fingolimod, a similar number of genes were differentially expressed by Mock- and HIV-exposed as compared to untreated cultures. However, among the genes differentially upregulated, the proportion of genes associated with immune responses was almost double in the HIV-exposed cultures (17/30, 57%) compared to Mock-exposed cultures (10/33, 30%). This HIV effect was reinforced by data from the pairwise comparison between HIV-exposed versus Mock-exposed gene expression. All nine genes that were upregulated (FC ≥ 1.5) were immune response genes. When FTY720-P was added daily to the differentiation medium, the number of genes that were differentially expressed increased by 33% in HIV-exposed and 40% in Mock-exposed cultures as compared to untreated cultures. However, fingolimod affected the gene expression differently, depending on the culture treatment. Genes involved in glycolysis were upregulated in the presence or absence of FTY720-P in Mock-exposed versus untreated cultures. However, in HIV-exposed versus untreated cultures, upregulation of glycolysis-related genes was only significant in the presence of FTY720-P, not in its absence. Expression of genes related to immune responses increased in Mock-exposed and even more so in HIV-exposed neurons compared to untreated cultures. Fingolimod depressed the magnitude of this response but only in HIV-exposed neurons. These observations from the differentially expressed gene sets were reinforced by functional enrichment analysis. The top canonical pathways and GO processes were dominated by glycolysis and immune response functions in either Mock-exposed or HIV-exposed cultures. The number of genes enriching these pathways or processes was increased in the FTY720-P-treated cultures. When examining the direct effect of fingolimod on cellular pathways and processes within a single culture treatment, i.e., comparing FTY720-P versus no FTY720-P treatment, four canonical pathways were found to be significantly affected by fingolimod in all three culture treatments. The only gene enriching the pathways was FST, the inhibitor of activin-mediated signaling, which was downregulated by FTY720-P in all culture treatments. GO processes related to signaling or immune responses were significantly affected by fingolimod in all three culture treatments. But distinct genes enriched these processes for each culture treatment: among others, HLA-A, HLA-B, APP, and AP2B1 for HIV cultures; ANXA1 and FST for Mock-exposed cultures; and annexin I and the transcriptional regulators ID1, ID2, and ID3 for untreated cultures.

Clinical trial study extensions have shown that a reduced rate of brain volume loss is sustained in patients with relapsing MS receiving fingolimod continuously (De Stefano et al. 2017). This clinical result may reflect a direct neuroprotective effect of fingolimod in the CNS. Neuroprotection could be enabled by the cellular S1P receptors present on astrocytes, microglia, and neurons, as well as the drug’s accessibility through the blood–brain barrier (Foster et al. 2007). A role for astrocytes has been demonstrated in studies using both animal and cell culture models. In the rodent model of MS, experimental autoimmune encephalomyelitis (EAE), conditional null mouse mutants lacking S1P1 on GFAP-expressing astrocytes but not on neurons showed attenuation of both EAE disease and FTY720-P efficacy (Choi et al. 2011). In an astroglial cell culture model, addition of tumor necrosis factor-alpha (TNF-α) and FTY720-P to human primary embryonic astrocytes and astrocytoma cultures was used to mimic an inflammatory milieu in the CNS. Fingolimod treatment resulted in changes in astroglial gene expression, specifically, the induction of mRNA for neuroprotective factors leukemia inhibitory factor (LIF), interleukin 11 (IL11), and heparin-binding EGF-like growth factor (HBEGF), and the inhibition of TNF-induced inflammatory genes (CXCL10, BAFF, MX1, and OAS2) (Hoffmann et al. 2015). Our study also provides evidence that FTY720-P treatment might be directly neuroprotective in inflammatory milieus that may be toxic or detrimental to neurons. In the neuronal lineage-specific cell culture used in this study, differential gene expression is not confounded by the presence of other neural cell lineages and thus reflects the direct effect of fingolimod on neurons. Therefore, fingolimod’s neuroprotective potential may be inferred from its observed pleotropic effects on neuronal gene expression related to activin signaling, glucose metabolism, immune responses, and amyloid-β production.

Fingolimod treatment has a potentially neuroprotective effect through the differential down regulation in neurons of the gene for follistatin, a major antagonist of activin signaling. Specifically, FTY720-P treatment significantly downregulated follistatin gene expression in all culture treatments, albeit with slightly different FC values among the three culture treatments (untreated, Mock-exposed, HIV-exposed). Follistatin is an antagonist of several TGF-beta family members, mainly activin A. Follistatin binds activin A with great affinity and targets it for lysosomal degradation, thus neutralizing its biological function (Hedger et al. 2011). Activin A has multiple neurotrophic effects. In chicken embryos, activin A stimulates and follistatin inhibits neurite outgrowth in primary cultures from dorsal root ganglia (Fang et al. 2012) but does not inhibit nerve growth factor-induced growth. In an experimental mouse model of kainic acid-induced brain injury, the protective effects of basic fibroblast growth factor (bFGF) were mediated through activin A. The abolishment of activin A function by follistatin resulted in excitotoxic cell death (Tretter et al. 2000). Rat spiral ganglion neurons incubated with activin A, brain-derived neurotrophic factor (BDNF), and erythropoietin had significantly improved survival and neurite outgrowth as compared to control cultures not containing these factors (Kaiser et al. 2013). Even though the gene for activin A itself is not differentially upregulated by FTY720-P in any culture conditions, the reduction in the mRNA for its inhibitor, follistatin, can indirectly result in increased neurotrophic activin actions.

Analyses of both functionally enriched canonical pathways and differentially expressed gene sets indicate that differential gene expression related to neuronal glucose utilization is enhanced by fingolimod treatment. In the comparison of Mock-exposed versus untreated cultures, upregulation was significant for 11 glycolysis-related genes in the presence of FTY720-P and for nearly half those genes in the absence of FTY720-P. In the comparison of HIV-exposed versus untreated cultures, however, this upregulation was significant only in the presence of the drug and not in its absence for all genes examined. This implies that HIV exposure alone does not significantly enhance glycolysis-related gene expression compared to that in untreated cultures, while Mock exposure does. The functional link between FTY720-P treatment and more robust glucose metabolism in the HIV-exposed neurons may be through signaling pathways that FTY720 is known to affect. In particular, there may be a role for the Akt-mediated survival pathways in neurons. Fingolimod activates ERK and Akt signaling pathways through phosphorylation of the ERK1/ERK2 or Akt substrates, something we observed with the hNP1 cultures (data not shown). Akt signaling is linked to upregulation of glucose metabolism in proliferating cells and in particular in cancer cells (Simons et al. 2012). Akt has been shown to be activated by FTY720 in rodent brain (Ren et al. 2017; Zhang et al. 2016) and human neuroblastoma cells (Ren et al. 2017) under conditions of stress such as traumatic brain injury, illustrated in the rodent model (Zhang et al. 2016). Akt may increase expression of glycolysis genes through inhibition of transcription factors that otherwise repress the expression of glycolysis genes (Simons et al. 2012). Moreover, energy utilization is of paramount importance in the protection of neurons. In neurodegenerative diseases such as Alzheimer’s, low glucose metabolism has been associated with cognitive decline (Furst et al. 2012). In multiple sclerosis where axonal demyelination is one of the primary hallmarks of the disease, neurons increase energy consumption in order to restore axonal impulse conduction (Trapp and Stys, 2009). A recent study of human CNS-derived oligodendrocytes (Rone et al. 2016) highlights the role of glycolysis in energy production by cells under metabolic stress. Stress-induced reduction in glycolytic ATP production can exacerbate myelin process withdrawal and compromise metabolic support of neurons.

An increasing body of evidence goes against the notion that neurons are immunosilent; on the contrary, neuronal immune responses can play a significant role in defense against pathogens, whether they are neurons directly infected by neurotropic viruses (see review by Chakraborty et al. (2010) or uninfected neurons in an inflammatory milieu (Boulanger and Shatz 2004). Fingolimod may modulate neuronal immune-related gene expression in an inflammatory milieu, leading to a net neuroprotective effect. In the HIV exposure paradigm of this study, the differentiating hNP1 cells are exposed to HIV proteins such as tat, gp120, nef, and vpr that can be neurotoxic (reviewed by Mocchetti et al. (2012). In the presence or absence of FTY720-P, exposure to Mock and HIV supernatants significantly upregulated genes associated with immune responses, as compared to untreated cultures (Fig. 7a, b). Even comparing HIV-exposed to Mock-exposed cultures, differential gene expression is significant for a number of MHC class I antigen-presenting genes as well as beta-2-microglobulin (B2M) (Fig. 7c). Consistent with the neuronal expression profile, MHC class II genes were not upregulated. With or without FTY720-P treatment, there are similar fold changes for the pairwise comparison of Mock-exposed versus untreated cultures (Fig. 7b). However, fold change values trend lower for the pairwise comparison of HIV-exposed versus untreated cultures in the presence of FTY720-P (Fig. 7a). Among the differentially expressed immune genes, B2M expression is 33% lower in the HIV-exposed hNP1 cultures treated with FTY720-P. Beta2-microglobulin, a component of MHC class I molecules, is reportedly elevated in the CSF of patients with HIV-associated dementing illnesses and in patients with Alzheimer’s disease (Brew et al. 1996; Carrette et al. 2003). The active yet lower immune gene expression in HIV-exposed hNP1 neurons presents a potential for neuroprotective consequences of FTY720-P treatment. Fingolimod’s net effect on the immune gene expression would be to lower the intensity of inflammation that could otherwise be detrimental to neurons in chronic HIV infection and aging (Canizares et al. 2014).

Further potential for fingolimod-mediated neuroprotection is reflected in the downregulating effect of FTY720-P on the APP gene and APP protein expression in HIV-exposed neurons. This depressive effect, though modest, was significant. Moreover, together with interferon-related and MHC I antigen-presenting genes, APP mRNA was downregulated by FTY720-P treatment significantly but only in the context of HIV exposure. This suggests an interaction between the expression of the APP gene and the inflammatory milieu created by HIV exposure of the neurons. APP is cleaved by β- and γ-secretases resulting in the production of amyloid-β peptides (De Strooper et al. 2010). Aggregation and plaque formation by amyloid-β constitute one of the defining pathological findings in the brain of Alzheimer’s disease patients (Blennow et al. 2006). In HIV-infected individuals, cerebral amyloid-β plaques predict HAND (Soontornniyomkij et al. 2012).

S1P, an essential component of the membrane lipid bilayer, has been found to have profound effects on neurodegeneration by modulating the cleavage of APP into amyloid-β protein among other possible pathways (reviewed by van Echten-Deckert et al. (2014)). Neurotoxicity mediated by amyloid-β protein was found to be ameliorated in primary mouse cortical neurons by fingolimod through upregulation of BDNF (Doi et al. 2013). An additional study, also using primary mouse cortical neurons, suggests that fingolimod can reduce amyloid-β protein production by a mechanism that is independent of the downstream SP1 receptor signaling pathways (Takasugi et al. 2013). In our study, expression of BDNF was not altered by addition of FTY-720 (data not shown), but protection against neurotoxicity mediated by amyloid-β might be achieved by a reduction of APP production.

This study adds to the body of evidence indicating that fingolimod can act directly on human neurons to modulate cell proliferation and survival. This study has the limitations inherent to the cell culture system to the extent that human neuronal cell lines can reflect the response of primary human neurons. In addition, technical detection of differentially regulated genes is limited by the number of probes included in the gene expression microarray platform and in the knowledge base of the software used for functional genomic analyses. But, given the study limitations, the data indicate that fingolimod has the capacity to alter neuronal gene expression in an inflammatory milieu, potentiating cellular responses that can have a net neuroprotective effect. That capacity is demonstrated here in the viral-specific inflammatory milieu generated by HIV exposure of the neurons. Through modest but significant downregulation of APP gene expression, fingolimod could protect against brain atrophy and cognitive decline associated with amyloid-β accumulation in chronic HIV infection. The drug fingolimod itself, as used in current neurological practice, is very problematic for the treatment of HIV infection or HAND since fingolimod’s actions on lymphocyte trafficking lead to a dramatic reduction in the number of circulating CD4-positive lymphocytes. But this study identifies neuroprotective avenues that can be targets for more specific S1P analogue development, particularly with respect to protection against amyloid-β accumulation. Thus, S1P signaling and related cell interactions can be exploited to treat and/or prevent neurodegenerative disease states such as HAND that are associated with chronic inflammation.

## Electronic supplementary material

ESM 1 (PDF 346 kb).
