# Supplementary material for: Fingolimod induces neuronal-specific gene expression with potential neuroprotective outcomes in maturing neuronal progenitor cells exposed to HIV
Source: J Neurovirol. 2017 Sep 14;23(6):808–24. doi: 10.1007/s13365-017-0571-7 (PMC5725524; doi:10.1007/s13365-017-0571-7)
Supplement: Supplementary file 1 — (PDF 346 kb) [file 13365_2017_571_MOESM1_ESM.pdf]

Fig. S1 Study design and experimental flow

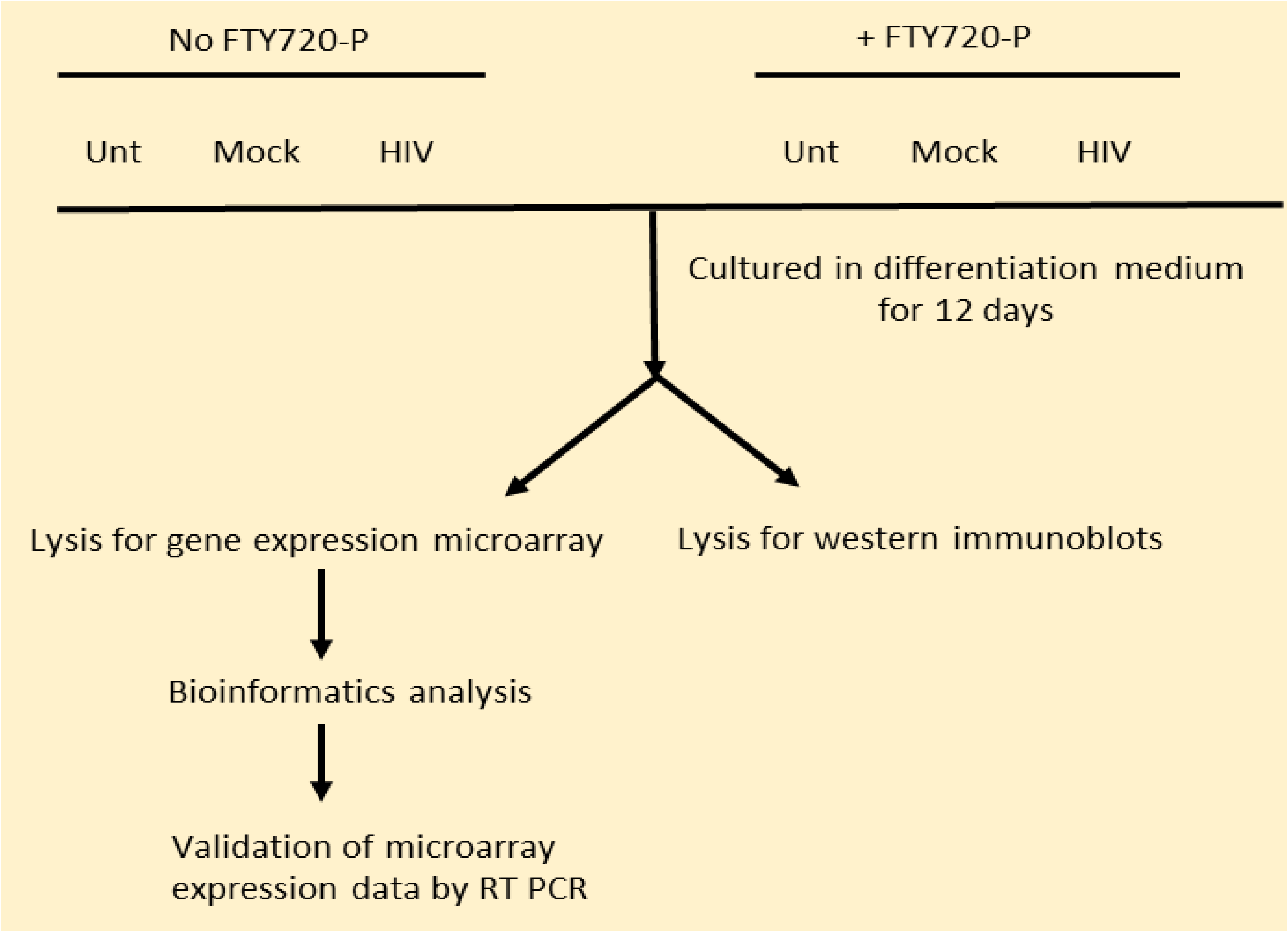

Table S1 Differentially expressed genes in pairwise comparisons of Mock vs Unt, HIV vs Unt and HIV vs Mock, in the absence of FTY720-P

NOTE: For all the analyses, repeated probes, non coding RNA, withdrawn genes and pseudogenes were removed

Expression values for antisense RNA, and for uncharacterized genes were kept

FC absolute value ≥ 1.5 and nominal p value ≤0.05

Mock vs Unt - No FTY720-P

Gene	Fold Change	Nominal p value
A2M	6.5396	0
ADA	1.5569	0.0037
ADM	3.2333	0.011
AGTPBP1	1.573	0.0061
ALDOC	2.1583	0.0442
ALPK2	5.0277	0.0011
ANGPT1	-1.6337	0.0015
ANKRD20A1	-1.6495	0.0113
ANKRD37	1.5049	0.027
ARHGEF6	-1.6235	0.0303
ARL16	-1.5803	0.0193
ASGR1	1.6872	0.0088
B2M	4.0348	0.0001
BAALC	1.5232	0
BCAT1	1.5627	0.0004
BCL3	1.6435	0.0011
BCL6	1.6557	0.0001
BHLHB2	2.0881	0.0176
BLZF1	-1.5698	0.0067
C1QTNF6	2.1006	0.0002
C21ORF24	-1.6234	0.0236
C21ORF62	1.5688	0.0172
C2ORF80	1.6621	0.0058
C9ORF80	-1.5568	0.0182
CAPN5	1.5823	0.0055
CCDC125	-1.5449	0.025
CCND1	-1.6254	0.0055
CD44	1.5417	0.0122
CDK6	-1.5816	0.0035
CDKN1A	-1.5259	0.0256
CEBPD	1.5086	0.0466
COL1A2	1.5923	0.0163
CRCP	-2.024	0.0165
CRY1	1.5564	0.0033
CSF2RA	-1.6185	0.0059
CXCR7	1.8378	0.0002

HIV vs Unt - No FTY720-P

Gene	FC value	Nominal p value
A2M	6.7048	0.0105
AGTPBP1	1.5352	0.0485
ALPK2	5.849	0.0052
ARHGEF6	-1.5094	0.0334
B2M	7.1211	0.0002
BCAT1	1.73	0.0003
BCL6	1.5148	0.0078
BHLHE22	-1.7009	0.0328
C1QTNF6	1.763	0.0006
C21ORF62	1.5419	0.0022
CAPN5	1.5516	0.002
CNTNAP1	1.5086	0.0152
COL1A2	1.511	0.0099
CXCR7	1.6421	0.0136
D4S234E	-1.5103	0.0266
EFR3A	1.5448	0.0282
ELAVL2	-1.6194	0.0252
ELMOD1	2.9402	0.0014
EPHA4	-1.6583	0.0081
FAM167A	3.563	0.0001
FAM46A	3.0055	0.0073
FLRT3	-1.9597	0.0243
FMN2	1.5284	0.0004
FXYD5	1.5858	0.0009
GYG2	2.2503	0.0142
HLA-A	3.2445	0.0001
HLA-A29.1	2.5003	0.002
HLA-B	3.6293	0.0001
HLA-E	2.501	0.0045
HLA-F	2.4328	0.0006
HS.489254	1.7366	0.0131
HS.551307	-1.581	0.0228
HS3ST3A1	1.8762	0.0089
HSPE1	1.8864	0.0465
HTRA1	2.2216	0.001
IFI6	1.5658	0.0162

HIV vs Mock - No FTY720-P

Gene	FC value	Nominal p value
B2M	1.7648	0.0101
HLA-A	1.8762	0.0008
HLA-A29.1	1.5317	0.0138
HLA-B	2.3674	0.0008
HLA-E	1.577	0.022
HLA-F	1.6636	0.0045
PSMB8	1.5266	0.0061
PSMB9	2.1464	0.0491
TAP1	3.146	0.0161

Unique to Mock vs Unt	Unique to HIV vs Unt	Common to both
ADA	BHLHE22	A2M
ADM	CNTNAP1	AGTPBP1
ALDOC	D4S234E	ALPK2
ANGPT1	ELAVL2	ARHGEF6
ANKRD20A1	HLA-F	B2M
ANKRD37	HS.551307	BCAT1
ARL16	JAG1	BCL6
ASGR1	LOC100008589	C1QTNF6
BAALC	LOC645323	C21ORF62
BCL3	LOC653111	CAPN5
BHLHB2	NBPF20	COL1A2
BLZF1	PCDH18	CXCR7
C21ORF24	PSMB8	EFR3A
C2ORF80	PSMB9	ELMOD1
C9ORF80	PSME1	EPHA4
CCDC125	PTRF	FAM167A
CCND1	SOX9	FAM46A
CD44	SP8	FLRT3
CDK6	TNFRSF1A	FMN2
CDKN1A		FXYD5
CEBPD		GYG2
CRCP		HLA-A
CRY1		HLA-A29.1
CSF2RA		HLA-B
DACT2		HLA-E
DDIT4		HS.489254
DEM1		HS3ST3A1
DMC1		HSPE1
EID2B		HTRA1
ENO1		IFI6
FABP5		IFITM1
FAM162A		IFITM2
FLJ44124		IFITM3
FRAS1		IGFBP5
GJA1		IL13RA1
GRM3		IRF1

DACT2	1.5502	0.0013	IFITM1	4.1549	0.0032	HK2	IRF9
DDIT4	1.6928	0.0319	IFITM2	2.6421	0.0002	HRASLS3	ISG15
DEM1	-1.5079	0.0198	IFITM3	3.5539	0.0031	HSPB1	KBTBD8
DMC1	-1.5026	0.038	IGFBP5	1.8596	0.0084	ID3	KCNIP4
EFR3A	1.5259	0.0368	IL13RA1	1.828	0.0015	IDH1	LARGE
EID2B	-1.7017	0.0326	IRF1	2.5481	0.0126	IFI16	LEF1
ELMOD1	3.5279	0.0006	IRF9	3.2923	0.001	IGDCC4	LGALS3BP
ENO1	1.6816	0.0361	ISG15	2.749	0.005	IGFBP2	LOC100134006
EPHA4	-1.5661	0.0101	JAG1	-1.5323	0.0333	LDHA	LOC399939
FABP5	1.6688	0.0137	KBTBD8	1.7438	0.0001	LILRB1	LOC729384
FAM162A	1.6151	0.0098	KCNIP4	5.4885	0	LOC100132727	MEGF10
FAM167A	3.6045	0	LARGE	-1.5354	0.0087	LOC100190938	NCAM1
FAM46A	2.1784	0.004	LEF1	-1.5422	0.0212	LOC255783	NEUROG2
FLJ44124	-1.5686	0.016	LGALS3BP	1.8874	0.0152	LOC392437	NPPC
FLRT3	-1.6956	0.0068	LOC100008589	-1.895	0.0389	LOC440934	PARP9
FMN2	1.6251	0.017	LOC100134006	-1.9366	0.0002	LOC642362	PCDH17
FRAS1	-1.5146	0.0273	LOC399939	-1.7606	0.0013	MAB21L2	PDE1A
FXYD5	1.7013	0.001	LOC645323	-1.6191	0.0214	MIF	PEG10
GJA1	1.5888	0.0275	LOC653111	-1.6347	0.0148	MOBKL2B	PFKP
GRM3	-1.6711	0.0006	LOC729384	-1.8293	0.0039	MTP18	PHCA
GYG2	1.92	0.0041	MEGF10	-1.7874	0.0054	MYCN	PLEKHA2
HK2	1.9563	0.0312	NBPF20	-1.6205	0.0261	NKX6-2	PPP1R14C
HLA-A	1.7293	0.001	NCAM1	-1.5629	0.0367	OLFM1	PRSS23
HLA-A29.1	1.6324	0.0015	NEUROG2	-1.6035	0.0016	P4HA1	PSRC1
HLA-B	1.533	0.0031	NPPC	-1.7888	0.004	PCDH19	RALYL
HLA-E	1.5859	0	PARP9	2.1567	0.0229	PFKFB3	RBMS1
HRASLS3	1.5252	0.0042	PCDH17	-1.6178	0.0245	PGAM1	RHOBTB3
HS.489254	1.6389	0.0228	PCDH18	-1.5651	0.0341	PGAM4	RND3
HS3ST3A1	2.2142	0.0089	PDE1A	-1.6125	0.0119	PGK1	SERPINB6
HSPB1	1.5511	0.0054	PEG10	1.6396	0.0269	PHYHIPL	SERPING1
HSPE1	1.6807	0.0378	PFKP	1.8191	0.0024	PI15	SHISA2
HTRA1	2.6796	0.0002	PHCA	1.7252	0.0071	PLOD2	SLC2A3
ID3	1.689	0.0164	PLEKHA2	1.9844	0.0007	PLXDC2	SMAD6
IDH1	1.6271	0.0181	PPP1R14C	1.8848	0.0007	PPP1R3C	SNCAIP
IFI16	1.5495	0.0029	PRSS23	1.9409	0.0022	PPP2R2B	SOCS3
IFI6	1.5634	0.023	PSMB8	1.7666	0.0056	PRPH	SOSTDC1
IFITM1	4.7562	0.0002	PSMB9	2.4671	0.0367	PTPRD	SPAG16
IFITM2	2.8637	0.0009	PSME1	1.5176	0.0172	RARRES2	SPRYD5
IFITM3	3.7441	0.0053	PSRC1	2.0754	0.0144	RAXL1	SRGAP3
IGDCC4	1.5495	0.0011	PTRF	1.8483	0.0393	SFRP1	STAT1
IGFBP2	1.654	0.0084	RALYL	1.5671	0.0009	SORT1	SULF1
IGFBP5	1.9491	0.0014	RBMS1	1.8964	0.0028	SOX3	TAP1
IL13RA1	2.0309	0.0001	RHOBTB3	1.8253	0.0018	SYT7	TMEM158
IRF1	1.693	0.0012	RND3	-1.6701	0.0004	TMEFF2	TPST2

IRF9	2.2178	0.0011
ISG15	2.1556	0.0034
KBTBD8	1.9122	0.0041
KCNIP4	7.1305	0.0003
LARGE	-1.5554	0.0045
LDHA	1.6507	0.0246
LEF1	-1.5494	0.0335
LGALS3BP	1.8316	0.0024
LILRB1	-1.5039	0.0106
LOC100132727	-1.5189	0.0483
LOC10013400€	-1.8955	0.0044
LOC10019093ξ	-1.5672	0.0128
LOC255783	1.584	0.0004
LOC392437	1.6667	0.0076
LOC399939	-1.6837	0.0036
LOC440934	-1.6835	0.0098
LOC642362	-1.6812	0.005
LOC729384	-1.5939	0.0019
MAB21L2	-1.6019	0.0236
MEGF10	-1.7371	0.0024
MIF	1.7649	0.0076
MOBKL2B	1.654	0.0004
MTP18	1.884	0.0005
MYCN	1.5914	0.0056
NCAM1	-1.5475	0.0105
NEUROG2	-1.6098	0.0031
NKX6-2	2.4054	0.001
NPPC	-2.0614	0.0024
OLFM1	-1.5055	0.0331
P4HA1	1.9163	0.0362
PARP9	1.7401	0.0042
PCDH17	-1.8137	0.0018
PCDH19	-1.6199	0.0058
PDE1A	-1.6874	0.0302
PEG10	1.7976	0.0139
PFKFB3	1.5656	0.0169
PFKP	1.5641	0.0091
PGAM1	2.1598	0.0152
PGAM4	1.6115	0.0149
PGK1	1.6237	0.0242
PHCA	1.5845	0.012
PHYHIPL	-1.5425	0.002
PI15	-1.5808	0.0015
PLEKHA2	2.3799	0.0033

SERPINB6	2.0581	0.0001
SERPING1	1.5913	0.011
SHISA2	-1.6341	0.0259
SLC2A3	2.4483	0.0443
SMAD6	1.687	0.0016
SNCAIP	-1.8056	0.0003
SOCS3	1.8648	0.0072
SOSTDC1	1.6706	0.0085
SOX9	-1.591	0.0105
SP8	-1.5219	0.017
SPAG16	-1.6269	0.0128
SPRYD5	-2.0677	0.0085
SRGAP3	-1.5117	0.0215
STAT1	3.624	0.024
SULF1	1.7949	0.0128
TAP1	6.6548	0.0067
TMEM158	3.8987	0.0096
TNFRSF1A	1.5	0.0003
TPST2	1.5372	0.004
TRIM48	-2.3047	0.0069
TSPO	1.5605	0.015
VGF	2.2278	0.0319
ZC3HAV1	2.2043	0.0005

TPI1
VGLL3
ZNF738

TRIM48
TSPO
VGF
ZC3HAV1

PLOD2	1.9843	0.0334
PLXDC2	1.6449	0.0004
PPP1R14C	2.2475	0.0136
PPP1R3C	1.5195	0.0101
PPP2R2B	1.5236	0.0048
PRPH	2.0654	0.047
PRSS23	2.3574	0.0051
PSRC1	2.191	0.0099
PTPRD	-1.6159	0.0141
RALYL	1.8613	0.003
RARRES2	1.538	0.0034
RAXL1	-1.5017	0.0312
RBMS1	1.9471	0.0004
RHOBTB3	1.5868	0.0014
RND3	-2.0557	0.0013
SERPINB6	2.3447	0.0001
SERPING1	1.7213	0.0016
SFRP1	1.5198	0.0057
SHISA2	-1.6113	0.0028
SLC2A3	3.3889	0.0019
SMAD6	1.6698	0.0074
SNCAIP	-1.8788	0.0039
SOCS3	1.9355	0.0001
SORT1	1.5491	0.001
SOSTDC1	1.8693	0.0007
SOX3	-1.5166	0.0227
SPAG16	-1.591	0.0046
SPRYD5	-1.8818	0.0075
SRGAP3	-1.5008	0.0238
STAT1	2.0168	0.0004
SULF1	2.0796	0.0016
SYT7	1.5429	0.0188
TAP1	2.1155	0
TMEFF2	-1.5376	0.0015
TMEM158	3.4331	0.0001
TPI1	1.5307	0.0182
TPST2	1.8181	0.0049
TRIM48	-1.8975	0.0006
TSPO	1.5288	0.0073
VGF	1.5804	0.0265
VGLL3	-1.7053	0.0125
ZC3HAV1	2.0697	0.0007
ZNF738	-1.5013	0.0403

Table S2 Differentially expressed genes in pairwise comparisons of Mock vs Unt and HIV vs Unt in the absence of FTY720-P

NOTE: For all the analyses, repeated probes, non coding RNA, withdrawn genes and pseudogenes were removed

Expression values for antisense RNA, and for uncharacterized genes were kept

FC absolute value ≥ 2.0 and nominal p value ≤ 0.05

Mock vs Unt no FTY720-P			HIV vs Unt no FTY720-P					
Gene	FC value	Nominal p value	Gene	FC value	Nominal p value	Unique to Mock	Unique to HIV	Common to both
KCNIP4	7.1305	0.0003	B2M	7.1211	0.0002	ADM	HLA-B	KCNIP4
A2M	6.5396	0	A2M	6.7048	0.0105	NKX6-2	HLA-A	A2M
ALPK2	5.0277	0.0011	TAP1	6.6548	0.0067	PLEKHA2	IRF1	ALPK2
IFITM1	4.7562	0.0002	ALPK2	5.849	0.0052	PRSS23	HLA-E	IFITM1
B2M	4.0348	0.0001	KCNIP4	5.4885	0	PPP1R14C	HLA-A29.1	B2M
IFITM3	3.7441	0.0053	IFITM1	4.1549	0.0032	HS3ST3A1	PSMB9	IFITM3
FAM167A	3.6045	0	TMEM158	3.8987	0.0096	PGAM1	HLA-F	FAM167A
ELMOD1	3.5279	0.0006	HLA-B	3.6293	0.0001	ALDOC	GYG2	ELMOD1
TMEM158	3.4331	0.0001	STAT1	3.624	0.024	C1QTNF6	VGF	TMEM158
SLC2A3	3.3889	0.0019	FAM167A	3.563	0.0001	BHLHB2	PARP9	SLC2A3
ADM	3.2333	0.011	IFITM3	3.5539	0.0031	SULF1	SPRYD5	IFITM2
IFITM2	2.8637	0.0009	IRF9	3.2923	0.001	PRPH	TRIM48	HTRA1
HTRA1	2.6796	0.0002	HLA-A	3.2445	0.0001	IL13RA1		SERPINB6
NKX6-2	2.4054	0.001	FAM46A	3.0055	0.0073	CRCP		IRF9
PLEKHA2	2.3799	0.0033	ELMOD1	2.9402	0.0014	RND3		PSRC1
PRSS23	2.3574	0.0051	ISG15	2.749	0.005	NPPC		FAM46A
SERPINB6	2.3447	0.0001	IFITM2	2.6421	0.0002			ISG15
PPP1R14C	2.2475	0.0136	IRF1	2.5481	0.0126			TAP1
IRF9	2.2178	0.0011	HLA-E	2.501	0.0045			ZC3HAV1
HS3ST3A1	2.2142	0.0089	HLA-A29.1	2.5003	0.002			STAT1
PSRC1	2.191	0.0099	PSMB9	2.4671	0.0367			
FAM46A	2.1784	0.004	SLC2A3	2.4483	0.0443			
PGAM1	2.1598	0.0152	HLA-F	2.4328	0.0006			
ALDOC	2.1583	0.0442	GYG2	2.2503	0.0142			
ISG15	2.1556	0.0034	VGF	2.2278	0.0319			
TAP1	2.1155	0	HTRA1	2.2216	0.001			

C1QTNF6	2.1006	0.0002	ZC3HAV1	2.2043	0.0005
BHLHB2	2.0881	0.0176	PARP9	2.1567	0.0229
SULF1	2.0796	0.0016	PSRC1	2.0754	0.0144
ZC3HAV1	2.0697	0.0007	SERPINB6	2.0581	0.0001
PRPH	2.0654	0.047	SPRYD5	-2.0677	0.0085
IL13RA1	2.0309	0.0001	TRIM48	-2.3047	0.0069
STAT1	2.0168	0.0004			
CRCP	-2.024	0.0165			
RND3	-2.0557	0.0013			
NPPC	-2.0614	0.0024			

Table S3 Differentially expressed genes in pairwise comparisons of HIV vs Unt: hNP1 cells compared to other published studies of neural cells or brain tissues
FC absolute value ≥ 2.0 and nominal p value ≤ 0.05

Genes differentially expressed in hNP1	FC value	Name	Function	Geffin et. al, NEP ApoE3/E3	Geffin et. al, NEP ApoE3/E3 and ApoE3/E4	Roberts et. al.	Masliah et.al.	Borjabad et. al.	Gelman et. al. Neo-striatum	Gelman et. al. Frontal lobe	Gelman et. al. White matter
B2M	7.12	beta-2-microglobulin	Immune response	U, FC= 1.65, p=0.003	U, FC=1.67, p=0.00			U	U		U
A2M	6.7	alpha-2-macroglobulin	Metabolism	NS, FC 1.15, p=0.428	NS, FC=-1.02, p=0.93	U					
TAP1	6.65	transporter 1, ATP Binding Cassette	Immune response	U, FC= 2.55, p=0.004	U, FC=2.37, p=0.00			U	U		U
ALPK2	5.85	alpha kinase 2	Enzyme	NS, FC=1.45, p=0.061	U, FC=1.42, p=0.012						
KCNIP4	5.49	potassium voltage-gated channel interacting protein 4	Neuronal function	NS, FC=1.50, p=0.092	U, FC=1.51, p=0.007			D			
IFITM1	4.15	interferon induced transmembrane protein 1	Immune response	U, FC=3.66, p=0.014	U, FC=3.49, p=0.0006		U	U	U		
TMEM158	3.9	transmembrane protein 158	Unknown	NS, F=1.22, p=0.172	U, FC=1.18, p=0.034						
HLA-B	3.63	Major Histocompatibility Complex, Class I, B	Immune response	U, F=4.27, p=0.000	U, FC=3.79, p=0.000			U	U		U
STAT1	3.62	Signal transducer and activator of transcription 1	Immune response	U, F=2.34, p=0.025	U, FC=2.0, p=0.0011		U	U	U	U	U
FAM167A	3.56	Family with sequence similarity 167 Member A	Cell signaling	NS, FC=-1.07, p=0.126	NS, FC=1.00, p=0.9773						
IFITM3	3.55	interferon induced transmembrane protein 3	Immune response	U, FC=2.36, p=0.016	U, FC=2.57, p=0.0001		U	U	U		

IRF9	3.29	interferon regulatory factor 9	Immune response	U, FC=1.62, p=0.013	U, FC=1.78, p=0.0001				U		
HLA-A	3.24	Major Histocompatibility Complex, Class I, A	Immune response	U, FC=2.06, p=0.022	U, FC=1.82, p=0.0060				U		U
FAM46A	3.01	Family with sequence similarity 46 Member A	Enzyme	NS, FC=1.14, p=0.116	U, FC 1.13, p=0.0100	U					
ELMOD1	2.94	ELMO domain containing 1	Cell signaling	NS, FC=1.14, p=0.116	NS, FC=1.12, p=0.1415			D		D	
ISG15	2.75	ISG15 ubiquitin-like modifier	Immune response	NS, FC=2.94, p=0.064	U, FC=3.66, p=0.0002		U	U	U	U	U
IFITM2	2.64	interferon induced transmembrane protein 2	Immune response	U, FC=2.51, p=0.016	U, FC=2.47, p=0.0003				U		
IRF1	2.55	interferon regulatory factor 1	Immune response	U, FC=1.68, p=0.019	U, FC=1.42, p=0.0111				U		
HLA-E	2.5	Major Histocompatibility Complex, Class I, E	Immune response	U, FC=2.34, p=0.002	U, FC=2.43, p=0.000		U	U	U		
HLA-A29.1	2.5	Major Histocompatibility Complex, Class I, A	Immune response	U, FC=2.17, p=0.050	U, FC=1.70, p=0.0418						
PSMB9	2.47	Proteasome submunit Beta 9	Immune response	U, FC=3.32, p=0.00	U, FC=1.62, p=0.0002			U	U		
SLC2A3	2.45	Solute Carrier Family 2 Member 3	Metabolism	NS, FC=1.14, p=0.084	NS, FC=1.08, FC=0.13					D	U
HLA-F	2.43	Major Histocompatibility Complex, Class I, F	Immune response	U, FC=2.28, p=0.001	U, FS=2.19, p=0.0001		U	U	U		U
GYG2	2.25	Glycogenin 2	Enzyme	NS, FC=1.10, p=0.251	NS, FC=1.03, p=0.69			U			
VGF	2.23	VGF Nerve Growth Factor Inducible	Neuronal function	NS, FC=-1.25, p=0.219	NS, FC=1.02, p=0.86						
HTRA1	2.22	HtrA serine peptidase 1	Enzyme	NS, FC=1.06, p=0.306	NS, FC=1.04, p=0.57						

ZC3HAV1	2.2	Zinc Finger CCCH-Type Containing, Antiviral 1	Immune response	NS, FC=1.18, p=0.093	U, FC=1.16, p=0.037	U	U	U	
PARP9	2.16	Poly(ADP-Ribose) Polymerase Family Member 9	Immune response	U, FC=1.68, p=0.019	U, FC=1.81, p=0.0002	U	U	U	U
PSRC1	2.08	Proline and Serine Rich Coiled-Coil 1	Metabolism	NS, FC=1.15, p=0.358	NS, FC=1.15, p=0.13	U	U		
SERPINB6	2.06	Serpin Family B Member 6	Metabolism	NC, FC=1.07, p=0.036	U, FC=1.26, p=0.0035	U			
SPRYD5	-2.07	Tripartite Motif-Containing (TRIM) 51	TRIM protein	NS, FC=-1.04, p=0.37	NS, FC=-1.33, p=0.90				
TRIM48	-2.3	Tripartite Motif-Containing (TRIM) 48	TRIM protein	NS, FC=-1.01, p=0.92	NS, FC=-1.0, p=0.39				

Genes differentially expressed in HIV-exposed hNP1 cultures and with |FC| values of 2.0 or more were compared to gene expression data from five published studies that investigated HIV exposure and infection of neural cells and tissues. The genes that are differentially expressed in the other studies but not in the hNP1-derived gene list are not included. In the data listed under Geffin et. al., are changes in gene expression that result from HIV exposure of differentiating human neuroepithelial progenitor cells (NEP) compared to untreated NEP. The gene expression data is stratified according to NEP that had only the apolipoprotein E (ApoE) E3/E3 genotype versus NEP that included both ApoE3/E3 and ApoE3/E4 genotypes. The data that is presented is from the microarray-generated data base of the Geffin et al, 2013 study; these are gene expression values that were analyzed but not published. Data under Roberts et. al., are genes differentially regulated in in the frontal lobe of SIV-infected macaques with encephalitis as compared to uninfected macaques. Masliah et al describes differences of gene expression in autopsied brains of infected vs non-infected individuals. Borbajabad et. al. studied gene expression in brains of individuals treated and not treated with anti-retroviral therapy. Listed are the comparisons of gene expression in brains tissues of untreated individuals with HAND as compared to uninfected controls. Data under Gelman et. al., are the genes differentially expressed in tissues from 3 brain regions in HIV-infected individuals with neurocognitive impairment and HIV encephalitis as compared to tissues from the same brain regions from uninfected individuals

U upregulated in this study, D downregulated in this study
Yellow highlight: Differentially regulated with |FC|>1.5 P<0.05
Blue highlight: Differentially regulated with |FC|>1.2 P<0.05

Table S4 Differentially expressed genes in pairwise comparisons of Mock vs Unt, HIV vs Unt and HIV vs Mock, in the presence of 10nM FTY720-P

Differentially expressed genes in pairwise comparisons of Mock vs Unt, HIV vs Unt and HIV vs Mock, in the presence of FTY720-P

NOTE: For all the analyses, repeated probes, non coding RNA, withdrawn genes and pseudogenes were removed

Expression values for antisense RNA, and for uncharacterized genes were kept

FC absolute value ≥ 1.5 and nominal p value ≤0.05

Mock vs Unt - with FTY720-P			HIV vs Unt - with FTY720-P			HIV vs Mock - with FTY720-P		
Gene	Fold Change	Nominal p value	Gene	FC value	Nominal p value	Gene	FC value	Nominal p value
A2M	7.2657	0	A2M	4.7322	0.4072	C21ORF24	1.5485	0.0481
ADA	1.5241	0.0008	ADM	3.5551	0.7388	CAPZB	-1.5783	0.0094
ADM	3.9414	0.0115	ALDOA	2.1877	0.4449	CATSPER2	1.5024	0.0442
AK3L1	1.6711	0.006	ALDOC	2.998	0.38	HLA-A	1.6016	0.0133
ALDOA	2.0984	0.0013	ALPK2	5.2758	0.3542	HLA-B	1.8217	0.0048
ALDOC	3.0076	0.0001	ANKRD37	1.7188	0.5287	OCIAD1	1.5899	0.0115
ALPK2	5.5056	0	AP2B1	-1.5313	0.7425	PALM	1.9108	0.001
ALPP	-1.653	0.0025	ARHGEF6	-1.5183	0.6351	SLC39A1	-1.6235	0.0013
ANKRD20A1	-1.6778	0.0053	B2M	4.9031	0.2422	STAT1	1.756	0.0457
ANKRD37	1.7605	0.0007	BCAT1	1.6865	0.5813	TAF15	-1.848	0.0132
ARRDC4	1.6262	0.0059	BCKDHA	1.5396	0.7831	TAP1	2.9136	0.0217
B2M	3.6721	0.0001	BCL6	1.6202	0.2362	WDR5	1.5041	0.0033
BAALC	1.589	0.0109	BNIP3	3.9014	0.2422	ZNF629	1.6209	0.0028
BCAT1	1.663	0.0029	C12ORF69	-1.8115	0.5796	ZNF682	1.5068	0.0321
BCL3	1.7433	0.0021	C1QTNF6	2.0108	0.4072			
BCL6	1.7452	0	C2ORF80	1.6548	0.2362			
BHLHB2	2.1962	0.0063	C8ORF13	3.2673	0.3423			
BLZF1	-1.6522	0.0117	CAPN5	1.7692	0.5228			
BNIP3	4.123	0.0002	CAPZB	-1.5847	0.5606			
C12ORF69	-1.5674	0.032	CDK6	-1.7457	0.6103			
C14ORF153	-1.5319	0.0015	CRY1	1.6903	0.6383			
C14ORF85	-1.5116	0.0299	CXCR7	1.9945	0.5489			
C15ORF63	-1.6295	0.0034	DDIT4	1.8296	0.662			
C1QTNF6	2.2352	0.0005	DTD1	1.684	0.5776			
C21ORF62	1.5448	0.0024	ELMOD1	2.7494	0.3423			
C2ORF80	1.9976	0.0001	ENC1	-1.5213	0.5287			
C8ORF45	-1.6612	0.0225	ENO1	1.8474	0.2362			
C9ORF80	-1.5296	0.0374	ENO2	1.7725	0.5083			
CA2	1.6528	0.0067	ERH	-1.5219	0.7113			
CAPN5	1.7316	0.0022	FAM162A	1.7706	0.5324			
CDK6	-2.0527	0.009	FAM46A	2.5217	0.2422			
CDKN2AIPNL	-1.6529	0.0257	FLJ35801	-1.5282	0.6851			
CEBPD	1.5309	0.0044	FLRT3	-1.601	0.7831			
CHRNA5	-1.6407	0.0021	FXYD5	1.6876	0.5083			

Unique to Mock vs Unt	Unique to HIV vs Unt	Common to both
ADA	AP2B1	A2M
AK3L1	ARHGEF6	ADM
ALPP	BCKDHA	ALDOA
ANKRD20A1	C8ORF13	ALDOC
ARRDC4	CAPZB	ALPK2
BAALC	DTD1	ANKRD37
BCL3	ERH	B2M
BHLHB2	FLJ35801	BCAT1
BLZF1	HLA-A29.1	BCL6
C14ORF153	HLA-B	BNIP3
C14ORF85	HLA-F	C12ORF69
C15ORF63	KIFC1	C1QTNF6
C21ORF62	LEF1	C2ORF80
C8ORF45	LMNB1	CAPN5
C9ORF80	LOC100008589	CDK6
CA2	LOC338758	CRY1
CDKN2AIPNL	PALM	CXCR7
CEBPD	PSMB8	DDIT4
CHRNA5	PSMD3	ELMOD1
CNTNAP1	PTRF	ENC1
COL1A2	RBM39	ENO1
CREB1	SPAG16	ENO2
CSF2RA	TAF15	FAM162A
CTDSP2		FAM46A
CYR61		FLRT3
DACT2		FXYD5
DDX51		GYG2
DEM1		HK1
DMC1		HK2
DOK5		HLA-A
DPY19L1		HLA-E
DUSP19		HS.489254
DUXAP3		HS3ST3A1
EBPL		HSPB1
EFNA1		HTRA1

CNTNAP1	1.5263	0.0165	GYG2	1.6306	0.3202	EID2B	ID3
COL1A2	1.621	0.003	HK1	1.5117	0.6567	EPHA4	IDH1
CREB1	-1.5849	0.0118	HK2	2.0146	0.4723	FABP5	IFI6
CRY1	1.7975	0.0008	HLA-A	2.6952	0.5207	FABP5L2	IFITM1
CSF2RA	-1.5267	0.0054	HLA-A29.1	2.1404	0.6351	FAM115A	IFITM2
CTDSP2	1.6178	0.0173	HLA-B	2.4434	0.4723	FAM167A	IFITM3
CXCR7	2.1824	0	HLA-E	2.2059	0.5813	FAM175A	IGDCC4
CYR61	-1.6944	0.0145	HLA-F	2.3966	0.5796	FBXO32	IGFBP2
DACT2	1.6502	0.007	HS.489254	1.7608	0.5134	FKBP14	IGFBP5
DDIT4	1.9579	0.0201	HS3ST3A1	1.5619	0.6334	FLJ44124	IL13RA1
DDX51	-1.7448	0.0034	HSPB1	1.6079	0.4723	FLJ46309	INSIG2
DEM1	-1.5023	0.0182	HTRA1	2.7397	0.3542	FMN2	IRF1
DMC1	-1.5591	0.0038	ID3	2.8935	0.3542	FRAS1	IRF9
DOK5	-1.5493	0.0006	IDH1	1.6352	0.6103	GAS2L3	ISG15
DPY19L1	1.5808	0.0022	IFI6	1.7802	0.2362	GRIPAP1	KBTBD8
DUSP19	-1.6888	0.0081	IFITM1	3.8248	0.5184	GRM3	KCNIP4
DUXAP3	-1.5709	0.0235	IFITM2	2.4071	0.4327	HCG2P7	LARGE
EBPL	1.5552	0.0049	IFITM3	3.5633	0.2422	HIATL2	LDHA
EFNA1	1.6075	0.0005	IGDCC4	1.5342	0.5861	HNRNPU	LGALS3BP
EID2B	-1.5698	0.0045	IGFBP2	1.533	0.794	HRASLS3	LOC100134006
ELMOD1	3.8597	0.0001	IGFBP5	1.954	0.5364	HRC	LOC399939
ENC1	-1.5496	0.0013	IL13RA1	1.9477	0.4072	HS.10862	LOC653111
ENO1	1.584	0.0121	INSIG2	1.5181	0.2362	HS.544637	LOC729384
ENO2	1.6421	0.0355	IRF1	1.9721	0.6705	HS.551847	MEGF10
EPHA4	-1.6332	0.0127	IRF9	3.0936	0.5207	HSD17B11	MGC4677
FABP5	1.543	0.0077	ISG15	2.3579	0.6959	HSPC268	MIF
FABP5L2	1.5454	0.0025	KBTBD8	1.8573	0.4449	IFI16	MOBKL2B
FAM115A	-1.6009	0.0101	KCNIP4	5.849	0.2422	INSM2	MTP18
FAM162A	1.9557	0.0191	KIFC1	-1.5218	0.4449	JMJD1A	NEUROG2
FAM167A	3.8058	0.0005	LARGE	-1.5117	0.4723	KCNH6	NKX6-2
FAM175A	-1.6283	0.0216	LDHA	2.1265	0.6013	LGI2	NPPC
FAM46A	2.2072	0.0022	LEF1	-1.5049	0.4723	LIMCH1	P4HA1
FBXO32	1.6354	0.0069	LGALS3BP	1.8479	0.2422	LOC100128098	PARP9
FKBP14	-1.5969	0.0243	LMNB1	-1.5705	0.4327	LOC100128505	PDE1A
FLJ44124	-1.6102	0.0287	LOC1000085	-2.781	0.5083	LOC100190938	PEG10
FLJ46309	-1.5248	0.0161	LOC1001340	-1.8275	0.6567	LOC202781	PFKFB3
FLRT3	-1.5228	0.0399	LOC338758	-1.5133	0.7644	LOC255783	PFKFB4
FMN2	1.5847	0.0008	LOC399939	-1.9858	0.3202	LOC399900	PFKP
FRAS1	-1.5404	0.0001	LOC653111	-1.7108	0.4984	LOC401098	PGAM1
FXYD5	1.5469	0.0018	LOC729384	-1.54	0.3423	LOC440934	PGAM4
GAS2L3	1.5649	0.0102	MEGF10	-1.794	0.589	LOC642362	PGK1
GRIPAP1	-1.6711	0.0242	MGC4677	1.7292	0.7459	LOC645895	PHCA
GRM3	-1.5477	0.0232	MIF	1.63	0.7117	LOX	PLEKHA2
GYG2	1.6726	0.0088	MOBKL2B	1.6791	0.5083	LRAP	PLOD2
HCG2P7	-1.5824	0.0069	MTP18	2.0099	0.6351	MCART1	PON2
HIATL2	-1.6117	0.0429	NEUROG2	-1.7712	0.6088	MCM8	PPP1R14C
HK1	1.5034	0.0331	NKX6-2	2.141	0.2834	NCRNA00219	PRPH

HK2	2.4524	0.0046	NPPC	-1.6308	0.7212	NET1	PRSS23
HLA-A	1.6828	0.0016	P4HA1	2.4626	0.4072	OCIAD1	PRSS35
HLA-E	1.5359	0.0018	PALM	1.9292	0.402	P4HA2	PSME1
HNRNPU	-1.6631	0.0169	PARP9	1.9444	0.5684	PDE4C	PSRC1
HRASLS3	1.8689	0.0048	PDE1A	-1.5121	0.5489	PGM1	RALYL
HRC	1.6828	0.0061	PEG10	1.5192	0.7055	PI15	RBMS1
HS.10862	1.5849	0.0095	PFKFB3	1.9163	0.6351	PIP5K2B	RHOBTB3
HS.489254	1.5289	0.0045	PFKFB4	2.514	0.6355	PLAGL1	RND3
HS.544637	-1.6535	0.0342	PFKP	1.8586	0.38	PLK2	RNU6-15
HS.551847	1.5895	0.0272	PGAM1	1.9184	0.2362	PLOD1	RPL12P6
HS3ST3A1	1.8431	0.0142	PGAM4	1.8269	0.5548	PNPT1	RPS29
HSD17B11	1.848	0.0009	PGK1	2.0037	0.5207	PPP1R3C	SERPINB6
HSPB1	1.9047	0	PHCA	1.7905	0.4156	PPP2R2B	SERPING1
HSPC268	-1.5497	0.0227	PLEKHA2	1.926	0.4072	PROS1	SLC16A3
HTRA1	3.3168	0.0005	PLOD2	2.698	0.535	PSMD12	SLC2A14
ID3	2.3943	0.0049	PON2	1.6232	0.5083	QRFPR	SLC2A3
IDH1	1.799	0.0021	PPP1R14C	1.999	0	S1PR3	SMAD6
IFI16	1.5574	0.0257	PRPH	1.643	0.2362	SFRP1	SNCAIP
IFI6	1.7423	0.0026	PRSS23	2.161	0.3202	SFRP2	SOCS3
IFITM1	5.0905	0.0001	PRSS35	-1.5207	0.4894	SH3BP4	SORT1
IFITM2	2.4929	0.0041	PSMB8	1.5325	0.6647	SHRM	SOSTDC1
IFITM3	3.9743	0.0008	PSMD3	1.5014	0.4449	SHROOM4	SPRYD5
IGDCC4	2.0386	0.0001	PSME1	1.5678	0.6632	SLC35E1	STAT1
IGFBP2	1.9123	0.0124	PSRC1	2.1505	0.2362	SLC44A4	STC1
IGFBP5	2.0441	0.0004	PTRF	1.7351	0.7551	SNORD13	SULF1
IL13RA1	2.0657	0.0011	RALYL	1.5339	0.4723	STAG3L2	TAP1
INSIG2	1.6591	0.0001	RBM39	-1.6305	0.6532	SULT1A1	TMEM158
INSM2	1.5623	0.0199	RBMS1	1.8447	0.6567	SYT7	TPI1
IRF1	1.6791	0	RHOBTB3	1.6031	0.7517	TIAM2	TPST2
IRF9	2.3075	0.001	RND3	-1.5604	0.7057	TIGA1	TRIM48
ISG15	1.8498	0.0001	RNU6-15	-1.5543	0.4156	TMEFF2	VKORC1
JMJD1A	1.5161	0.0045	RPL12P6	1.5846	0.7352	TNFRSF1A	ZC3HAV1
KBTBD8	2.2963	0.0003	RPS29	1.6164	0.7478	TSPO	
KCNH6	-1.6624	0.0046	SERPINB6	2.0819	0	UBE2L6	
KCNIP4	8.0289	0	SERPING1	1.5609	0.5427	VGLL3	
LARGE	-1.7306	0.0012	SLC16A3	1.9673	0.6632	XPNPEP3	
LDHA	2.4813	0.0138	SLC2A14	1.5765	0.2422	ZNF483	
LGALS3BP	1.7516	0.0001	SLC2A3	3.7218	0.2362	ZNF652	
LGI2	-1.5764	0	SMAD6	1.8051	0.2834	ZNF69	
LIMCH1	-1.6881	0.0186	SNCAIP	-1.5246	0.535		
LOC100128098	-1.5528	0.0289	SOCS3	2.0694	0.7425		
LOC100128505	-1.6507	0.0183	SORT1	1.6998	0.6088		
LOC100134006	-1.9003	0.0004	SOSTDC1	1.5215	0.5629		
LOC100190938	-1.7066	0.0079	SPAG16	-1.5168	0.6334		
LOC202781	-1.5904	0.0204	SPRYD5	-1.8457	0.6596		
LOC255783	1.6628	0.0024	STAT1	2.8999	0.583		
LOC399900	-1.6092	0.0076	STC1	1.71	0.4449		

LOC399939	-1.8869	0.0003	SULF1	1.5801	0.6148
LOC401098	-1.5614	0.0373	TAF15	-1.924	0.5861
LOC440934	-1.7339	0.0014	TAP1	5.0343	0.6334
LOC642362	-1.6598	0.0025	TMEM158	3.3193	0.6052
LOC645895	-1.5644	0.0001	TPI1	1.5958	0.6286
LOC653111	-1.8181	0.0003	TPST2	1.5709	0.6594
LOC729384	-1.846	0.0021	TRIM48	-2.071	0.3542
LOX	1.5471	0.0151	VKORC1	1.6016	0.6632
LRAP	-1.6261	0.0039	ZC3HAV1	1.996	0.4723
MCART1	-1.787	0.0188			
MCM8	-1.6856	0.0074			
MEGF10	-1.7751	0.0012			
MGC4677	1.5038	0.0002			
MIF	2.0347	0.0051			
MOBKL2B	1.8753	0.0043			
MTP18	1.9788	0.0004			
NCRNA00219	1.5574	0.0006			
NET1	1.842	0			
NEUROG2	-1.9586	0.0072			
NKX6-2	2.506	0.0001			
NPPC	-2.0823	0.0035			
OCIAD1	-1.7011	0.0451			
P4HA1	2.8741	0.0051			
P4HA2	1.5452	0.0361			
PARP9	1.6915	0.0004			
PDE1A	-1.6571	0.0028			
PDE4C	-1.6298	0.006			
PEG10	1.7509	0.006			
PFKFB3	1.6569	0.0005			
PFKFB4	2.6541	0.0005			
PFKP	1.6261	0.0131			
PGAM1	2.0047	0.0001			
PGAM4	1.782	0.0078			
PGK1	2.2185	0.0008			
PGM1	1.503	0.0122			
PHCA	1.7202	0.0035			
PI15	-1.8066	0.0038			
PIP5K2B	-1.5247	0.0328			
PLAGL1	-1.5814	0.0085			
PLEKHA2	2.2026	0.0028			
PLK2	1.5303	0.0026			
PLOD1	1.5757	0.0026			
PLOD2	2.5403	0.0023			
PNPT1	-1.7258	0.0084			
PON2	1.6422	0.0068			
PPP1R14C	2.566	0			
PPP1R3C	1.558	0.0012			

PPP2R2B	1.5289	0.0005
PROS1	1.761	0.0063
PRPH	2.3095	0
PRSS23	2.8574	0.0001
PRSS35	-1.5344	0.0001
PSMD12	-1.5215	0.0109
PSME1	1.5188	0.0192
PSRC1	2.2651	0.0004
QRFPR	-1.6904	0.015
RALYL	1.9465	0
RBMS1	2.0369	0.0004
RHOBTB3	1.617	0.0173
RND3	-1.6314	0.0017
RNU6-15	-1.5816	0.0062
RPL12P6	1.6248	0.0125
RPS29	1.6434	0.0098
S1PR3	-1.6063	0.0126
SERPINB6	2.2812	0
SERPING1	1.7698	0.008
SFRP1	1.5313	0.0002
SFRP2	-1.5815	0.0025
SH3BP4	1.5662	0.0018
SHRM	-1.5088	0.0386
SHROOM4	-1.6443	0.0164
SLC16A3	2.2052	0.0208
SLC2A14	1.7717	0.0011
SLC2A3	4.4463	0.0004
SLC35E1	-1.5866	0.0396
SLC44A4	-1.5333	0.049
SMAD6	1.981	0.0002
SNCAIP	-1.6223	0.0025
SNORD13	1.7369	0.0076
SOCS3	2.0159	0.0016
SORT1	1.6236	0.003
SOSTDC1	1.6669	0.001
SPRYD5	-1.9378	0.0003
STAG3L2	-1.506	0.0149
STAT1	2.1656	0.0018
STC1	1.8459	0.0022
SULF1	1.9506	0
SULT1A1	-1.617	0.0448
SYT7	1.7476	0.0059
TAP1	1.7279	0.001
TIAM2	-1.528	0.0018
TIGA1	1.5106	0.0009
TMEFF2	-1.7552	0.0146
TMEM158	2.8843	0

TNFRSF1A	1.5709	0.0055
TPI1	1.8287	0.0017
TPST2	1.8203	0.0055
TRIM48	-2.0973	0
TSPO	1.5183	0.0026
UBE2L6	1.5206	0.0034
VGLL3	-1.5109	0.0044
VKORC1	1.5687	0.0024
XPNPEP3	-1.6739	0.0006
ZC3HAV1	1.8523	0
ZNF483	-1.5463	0.0185
ZNF652	-1.537	0.0397
ZNF69	-1.5759	0.0087

Table S5 Differentially expressed genes in pairwise comparisons of Mock vs Unt, and HIV vs Unt, in the presence of 10nM FTY720-P

NOTE: For all the analyses, repeated probes, non coding RNA, withdrawn genes and pseudogenes were removed

Expression values for antisense RNA, and for uncharacterized genes were kept

FC absolute value ≥ 2.0 and nominal p value ≤0.05

HIV vs Unt +FTY720-P			Mock vs Unt +FTY720					
Gene	FC value	Nominal p value	Gene	FC value	Nominal p value	Unique to HIV	Unique to Mock	Common to both
A2M	4.7322	0.001	A2M	7.2657	0	TAP1	PPP1R14C	KCNIP4
ADM	3.5551	0.0264	ADM	3.9414	0.0115	HLA-A	PRPH	ALPK2
ALDOA	2.1877	0.0013	ALDOA	2.0984	0.0013	HLA-B	KBTBD8	B2M
ALDOC	2.998	0.0007	ALDOC	3.0076	0.0001	HLA-F	SLC16A3	A2M
ALPK2	5.2758	0.0006	ALPK2	5.5056	0	ISG15	PLEKHA2	BNIP3
B2M	4.9031	0.0002	B2M	3.6721	0.0001	HLA-E	BHLHB2	IFITM1
BNIP3	3.9014	0.0002	BHLHB2	2.1962	0.0063	HLA-A29.1	CXCR7	SLC2A3
C1QTNF6	2.0108	0.0009	BNIP3	4.123	0.0002	MTP18	IL13RA1	IFITM3
ELMOD1	2.7494	0.0005	C1QTNF6	2.2352	0.0005	LOC100008589	IGFBP5	ADM
FAM167A	3.1516	0.0005	CDK6	-2.0527	0.009		IGDCC4	TMEM158
FAM46A	2.5217	0.0002	CXCR7	2.1824	0		RBMS1	FAM167A
HK2	2.0146	0.0016	ELMOD1	3.8597	0.0001		MIF	IRF9
HLA-A	2.6952	0.0028	FAM167A	3.8058	0.0005		PGAM1	ALDOC
HLA-A29.1	2.1404	0.0085	FAM46A	2.2072	0.0022		CDK6	STAT1
HLA-B	2.4434	0.0018	HK2	2.4524	0.0046		NPPC	ID3
HLA-E	2.2059	0.0048	HTRA1	3.3168	0.0005			ELMOD1
HLA-F	2.3966	0.0047	ID3	2.3943	0.0049			HTRA1
HTRA1	2.7397	0.0006	IFITM1	5.0905	0.0001			PLOD2
ID3	2.8935	0.0006	IFITM2	2.4929	0.0041			FAM46A
IFITM1	3.8248	0.0027	IFITM3	3.9743	0.0008			PFKFB4
IFITM2	2.4071	0.0012	IGDCC4	2.0386	0.0001			P4HA1
IFITM3	3.5633	0.0002	IGFBP5	2.0441	0.0004			IFITM2
IRF9	3.0936	0.0028	IL13RA1	2.0657	0.0011			ALDOA
ISG15	2.3579	0.016	IRF9	2.3075	0.001			PRSS23
KCNIP4	5.849	0.0002	KBTBD8	2.2963	0.0003			PSRC1

LDHA	2.1265	0.0055
LOC100008589	-2.781	0.0024
MTP18	2.0099	0.0086
NKX6-2	2.141	0.0003
P4HA1	2.4626	0.0009
PFKFB4	2.514	0.0088
PGK1	2.0037	0.0028
PLOD2	2.698	0.0035
PRSS23	2.161	0.0004
PSRC1	2.1505	0.0001
SERPINB6	2.0819	0
SLC2A3	3.7218	0.0001
SOCS3	2.0694	0.0275
STAT1	2.8999	0.0049
TAP1	5.0343	0.0076
TMEM158	3.3193	0.0056
TRIM48	-2.071	0.0006

KCNIP4	8.0289	0
LDHA	2.4813	0.0138
MIF	2.0347	0.0051
NKX6-2	2.506	0.0001
NPPC	-2.0823	0.0035
P4HA1	2.8741	0.0051
PFKFB4	2.6541	0.0005
PGAM1	2.0047	0.0001
PGK1	2.2185	0.0008
PLEKHA2	2.2026	0.0028
PLOD2	2.5403	0.0023
PPP1R14C	2.566	0
PRPH	2.3095	0
PRSS23	2.8574	0.0001
PSRC1	2.2651	0.0004
RBMS1	2.0369	0.0004
SERPINB6	2.2812	0
SLC16A3	2.2052	0.0208
SLC2A3	4.4463	0.0004
SOCS3	2.0159	0.0016
STAT1	2.1656	0.0018
TMEM158	2.8843	0
TRIM48	-2.0973	0

NKX6-2
LDHA
SERPINB6
SOCS3
HK2
C1QTNF6
PGK1
TRIM48

Table S6 Differentially expressed genes in pairwise comparisons of Mock vs Unt in the presence and absence of of 10nM FTY720-P
Mock versus Untreated with FC absolute value > 2.0 and nominal p value <0.05

Mock vs Unt no FTY720-P			Mock vs Unt +FTY720					
Gene	FC value	Nominal p value	Gene	FC value	Nominal p value	Unique to no FTY720-P	Unique to FTY720-P	Common to both
KCNIP4	7.1305	0.0003	A2M	7.2657	0	HS3ST3A1	BNIP3	KCNIP4
A2M	6.5396	0	ADM	3.9414	0.0115	ISG15	P4HA1	A2M
ALPK2	5.0277	0.0011	ALDOA	2.0984	0.0013	TAP1	PFKFB4	ALPK2
IFITM1	4.7562	0.0002	ALDOC	3.0076	0.0001	SULF1	PLOD2	IFITM1
B2M	4.0348	0.0001	ALPK2	5.5056	0	ZC3HAV1	LDHA	B2M
IFITM3	3.7441	0.0053	B2M	3.6721	0.0001	CRCP	HK2	IFITM3
FAM167A	3.6045	0	BHLHB2	2.1962	0.0063	RND3	ID3	FAM167A
ELMOD1	3.5279	0.0006	BNIP3	4.123	0.0002		KBTBD8	ELMOD1
TMEM158	3.4331	0.0001	C1QTNF6	2.2352	0.0005		PGK1	TMEM158
SLC2A3	3.3889	0.0019	CDK6	-2.0527	0.009		SLC16A3	SLC2A3
ADM	3.2333	0.011	CXCR7	2.1824	0		CXCR7	ADM
IFITM2	2.8637	0.0009	ELMOD1	3.8597	0.0001		ALDOA	IFITM2
HTRA1	2.6796	0.0002	FAM167A	3.8058	0.0005		IGFBP5	HTRA1
NKX6-2	2.4054	0.001	FAM46A	2.2072	0.0022		IGDCC4	NKX6-2
PLEKHA2	2.3799	0.0033	HK2	2.4524	0.0046		RBMS1	PLEKHA2
PRSS23	2.3574	0.0051	HTRA1	3.3168	0.0005		MIF	PRSS23
SERPINB6	2.3447	0.0001	ID3	2.3943	0.0049		SOCS3	SERPINB6
PPP1R14C	2.2475	0.0136	IFITM1	5.0905	0.0001		CDK6	PPP1R14C
IRF9	2.2178	0.0011	IFITM2	2.4929	0.0041		TRIM48	IRF9
HS3ST3A1	2.2142	0.0089	IFITM3	3.9743	0.0008			PSRC1
PSRC1	2.191	0.0099	IGDCC4	2.0386	0.0001			FAM46A
FAM46A	2.1784	0.004	IGFBP5	2.0441	0.0004			PGAM1
PGAM1	2.1598	0.0152	IL13RA1	2.0657	0.0011			ALDOC
ALDOC	2.1583	0.0442	IRF9	2.3075	0.001			C1QTNF6
ISG15	2.1556	0.0034	KBTBD8	2.2963	0.0003			BHLHB2
TAP1	2.1155	0	KCNIP4	8.0289	0			PRPH
C1QTNF6	2.1006	0.0002	LDHA	2.4813	0.0138			IL13RA1

BHLHB2	2.0881	0.0176
SULF1	2.0796	0.0016
ZC3HAV1	2.0697	0.0007
PRPH	2.0654	0.047
IL13RA1	2.0309	0.0001
STAT1	2.0168	0.0004
CRCP	-2.024	0.0165
RND3	-2.0557	0.0013
NPPC	-2.0614	0.0024

MIF	2.0347	0.0051
NKX6-2	2.506	0.0001
NPPC	-2.0823	0.0035
P4HA1	2.8741	0.0051
PFKFB4	2.6541	0.0005
PGAM1	2.0047	0.0001
PGK1	2.2185	0.0008
PLEKHA2	2.2026	0.0028
PLOD2	2.5403	0.0023
PPP1R14C	2.566	0
PRPH	2.3095	0
PRSS23	2.8574	0.0001
PSRC1	2.2651	0.0004
RBMS1	2.0369	0.0004
SERPINB6	2.2812	0
SLC16A3	2.2052	0.0208
SLC2A3	4.4463	0.0004
SOCS3	2.0159	0.0016
STAT1	2.1656	0.0018
TMEM158	2.8843	0
TRIM48	-2.0973	0

STAT1

NPPC

Table S7 Differentially expressed genes in pairwise comparisons of HIV vs Unt in the presence and absence of of 10nM FTY720-P
HIV versus Untreated with FC absolute value > 2.0 and nominal p value <0.05

HIV vs Unt no FTY720-P			HIV vs Unt +FTY720-P					
Gene	FC value	Nominal p value	Gene	FC value	Nominal p value	Unique to no FTY720-P	Unique to FTY720-P	Common to both
B2M	7.1211	0.0002	A2M	4.7322	0.001	IRF1	BNIP3	KCNIP4
A2M	6.7048	0.0105	ADM	3.5551	0.0264	PSMB9	ADM	ALPK2
TAP1	6.6548	0.0067	ALDOA	2.1877	0.0013	GYG2	ALDOC	TAP1
ALPK2	5.849	0.0052	ALDOC	2.998	0.0007	VGF	ID3	B2M
KCNIP4	5.4885	0	ALPK2	5.2758	0.0006	ZC3HAV1	PLOD2	A2M
IFITM1	4.1549	0.0032	B2M	4.9031	0.0002	PARP9	PFKFB4	IFITM1
TMEM158	3.8987	0.0096	BNIP3	3.9014	0.0002	SPRYD5	P4HA1	SLC2A3
HLA-B	3.6293	0.0001	C1QTNF6	2.0108	0.0009		ALDOA	IFITM3
STAT1	3.624	0.024	ELMOD1	2.7494	0.0005		PRSS23	TMEM158
FAM167A	3.563	0.0001	FAM167A	3.1516	0.0005		NKX6-2	FAM167A
IFITM3	3.5539	0.0031	FAM46A	2.5217	0.0002		LDHA	IRF9
IRF9	3.2923	0.001	HK2	2.0146	0.0016		SOCS3	STAT1
HLA-A	3.2445	0.0001	HLA-A	2.6952	0.0028		HK2	ELMOD1
FAM46A	3.0055	0.0073	HLA-A29.1	2.1404	0.0085		C1QTNF6	HTRA1
ELMOD1	2.9402	0.0014	HLA-B	2.4434	0.0018		MTP18	HLA-A
ISG15	2.749	0.005	HLA-E	2.2059	0.0048		PGK1	FAM46A
IFITM2	2.6421	0.0002	HLA-F	2.3966	0.0047		LOC100008589	HLA-B
IRF1	2.5481	0.0126	HTRA1	2.7397	0.0006			IFITM2
HLA-E	2.501	0.0045	ID3	2.8935	0.0006			HLA-F
HLA-A29.1	2.5003	0.002	IFITM1	3.8248	0.0027			ISG15
PSMB9	2.4671	0.0367	IFITM2	2.4071	0.0012			HLA-E
SLC2A3	2.4483	0.0443	IFITM3	3.5633	0.0002			PSRC1
HLA-F	2.4328	0.0006	IRF9	3.0936	0.0028			HLA-A29.1
GYG2	2.2503	0.0142	ISG15	2.3579	0.016			SERPINB6
VGF	2.2278	0.0319	KCNIP4	5.849	0.0002			TRIM48
HTRA1	2.2216	0.001	LDHA	2.1265	0.0055			
ZC3HAV1	2.2043	0.0005	LOC100008589	-2.781	0.0024			
PARP9	2.1567	0.0229	MTP18	2.0099	0.0086			

PSRC1	2.0754	0.0144
SERPINB6	2.0581	0.0001
SPRYD5	-2.0677	0.0085
TRIM48	-2.3047	0.0069

NKX6-2	2.141	0.0003
P4HA1	2.4626	0.0009
PFKFB4	2.514	0.0088
PGK1	2.0037	0.0028
PLOD2	2.698	0.0035
PRSS23	2.161	0.0004
PSRC1	2.1505	0.0001
SERPINB6	2.0819	0
SLC2A3	3.7218	0.0001
SOCS3	2.0694	0.0275
STAT1	2.8999	0.0049
TAP1	5.0343	0.0076
TMEM158	3.3193	0.0056
TRIM48	-2.071	0.0006

Table S8 Enrichment analysis report, canonical pathways- Mock vs Unt with and without FTY720-P

Enrichment by Pathway Maps

					Mock vs Unt no FTY720-P				Mock vs Unt +10 nM FTY720-P			
#	Maps	Total	min p Value	Min FDR	p-value	FDR	In Data	Network Objects from Active Data	p-value	FDR	In Data	Network Objects from Active Data
1	Glycolysis and gluconeogenesis	86	4.987E-11	1.746E-08	2.503E-09	6.821E-07	13	G3P2, LDHA, TPI1, HXK2, ALDOC, PFKP, GLUT3, PGK1, ENO1, ALDOA, PGAM1, PGAM4, GPI	4.987E-11	1.746E-08	17	G3P2, LDHA, TPI1, HXK2, HXK1, ALDOC, ENO3, PFKP, GLUT3, PGK1, ENO1, PFKL, ALDOA, PGAM1, PGAM4, ENO2, GPI
2	Glycolysis and gluconeogenesis/ Human version	86	4.987E-11	1.746E-08	2.503E-09	6.821E-07	13	G3P2, LDHA, TPI1, HXK2, ALDOC, PFKP, GLUT3, PGK1, ENO1, ALDOA, PGAM1, PGAM4, GPI	4.987E-11	1.746E-08	17	G3P2, LDHA, TPI1, HXK2, HXK1, ALDOC, ENO3, PFKP, GLUT3, PGK1, ENO1, PFKL, ALDOA, PGAM1, PGAM4, ENO2, GPI
3	Immune response_IFN alpha/beta signaling pathway	24	2.668E-06	4.720E-04	2.668E-06	4.720E-04	6	IRF1, IFI6, IRF9, STAT1, IFNAR2, ISG15	2.584E-05	1.809E-03	6	IRF1, IFI6, IRF9, STAT1, IFNAR2, ISG15
4	Cell cycle_Regulation of G1/S transition (part 1)	38	3.464E-06	4.720E-04	3.464E-06	4.720E-04	7	p21, Cyclin D2, TGF-beta 2, PP2A regulatory, Cyclin D1, Cyclin D, CDK6	4.564E-05	2.904E-03	7	p21, TGF-beta 2, PP2A regulatory, Cyclin D1, SMAD3, Cyclin D, CDK6
5	Immune response_Oncostatin M signaling via JAK-Stat in mouse cells	20	8.120E-06	1.137E-03	3.475E-04	1.457E-02	4	Cyclin D1, SOCS3, TIMP1, STAT1	8.120E-06	1.137E-03	6	STAT3, VEGF-A, Cyclin D1, SOCS3, TIMP1, STAT1
6	Development_YAP/TAZ-mediated co-regulation of transcription	56	1.188E-05	1.164E-03	2.831E-03	5.510E-02	5	ID3, Cyclin D1, Lef-1, SOX9, CDK6	1.188E-05	1.164E-03	9	ID3, UFO, VEGF-A, Cyclin D1, Lef-1, SMAD3, CTGF, Cyr61, CDK6
7	Immune response_IL-6 signaling pathway via JAK/STAT	72	1.482E-05	1.164E-03	2.459E-04	1.335E-02	7	p21, IRF1, p300, SOCS3, STAT1, C/EBPdelta, CDK6	1.482E-05	1.164E-03	10	p21, STAT3, IRF1, IRS-1, VEGF-A, SOCS3, STAT1, mTOR, C/EBPdelta, CDK6
8	Development_Thrombopoetin signaling via JAK-STAT pathway	22	1.496E-05	1.164E-03	3.146E-05	2.644E-03	5	p21, TAP1 (PSF1), Cyclin D1, SOCS3, STAT1	1.496E-05	1.164E-03	6	p21, STAT3, TAP1 (PSF1), Cyclin D1, SOCS3, STAT1
9	Immune response_Oncostatin M signaling via JAK-Stat in human cells	22	1.496E-05	1.164E-03	3.146E-05	2.644E-03	5	Cyclin D1, SOCS3, TIMP1, STAT1, LIFR	1.496E-05	1.164E-03	6	STAT3, VEGF-A, Cyclin D1, SOCS3, TIMP1, STAT1

10	Development_WNT signaling pathway. Part 2	53	3.396E-05	2.644E-03	3.396E-05	2.644E-03	7	CD44, Tcf(Lef), WNT, Cyclin D1, NRCAM, Lef-1, ENC1	4.005E-04	1.219E-02	7	CD44, Tcf(Lef), VEGF-A, Cyclin D1, NRCAM, Lef-1, ENC1

Table S9 Enrichment analysis report canonical pathways- HIV vs Unt with and without FTY720-P

Enrichment by Pathway Maps					HIV vs Unt No FTY720-p				HIV vs Unt +10nM FTY720-P			
#	Maps	Total	min p Value	Min FDR	p-value	FDR	In Data	Network Objects from Active Data	p-value	FDR	In Data	Network Objects from Active Data
1	Glycolysis and gluconeogenesis	86	1.201E-12	2.474E-10	6.940E-02	3.267E-01	3	PFKP, GLUT3, GPI	1.201E-12	2.474E-10	16	G3P2, PFKP, GLUT3, LDHA, PGK1, TPI1, ENO1, PFKL, ALDOA, PGAM1, HXK2, HXK1, ALDOC, PGAM4, ENO2, ENO3
2	Glycolysis and gluconeogenesis/ Human version	86	1.201E-12	2.474E-10	6.940E-02	3.267E-01	3	PFKP, GLUT3, GPI	1.201E-12	2.474E-10	16	G3P2, PFKP, GLUT3, LDHA, PGK1, TPI1, ENO1, PFKL, ALDOA, PGAM1, HXK2, HXK1, ALDOC, PGAM4, ENO2, ENO3
3	Immune response_Antigen presentation by MHC class I, classical pathway	54	1.158E-07	4.851E-05	1.158E-07	4.851E-05	8	Tapasin, MHC Class I alpha chain, MHC class I, PSMB9, TAP1 (PSF1), PSME1, Beta-2-microglobulin, PSMB8(LMP7)	3.806E-06	3.136E-04	8	MHC Class I alpha chain, PSME2, MHC class I, PA28 (11S regulator), TAP1 (PSF1), PSME1, Beta-2-microglobulin, PSMB8(LMP7)
4	Immune response_IFN alpha/beta signaling pathway	24	1.167E-07	1.603E-05	5.370E-06	7.500E-04	5	IRF9, IRF1, IFI6, STAT1, ISG15	1.167E-07	1.603E-05	7	IRF9, IRF1, IFI6, STAT1, IFNAR2, PRMT1, ISG15
5	Development_WNT signaling pathway. Part 2	53	1.666E-06	3.489E-04	1.666E-06	3.489E-04	7	NRCAM, CD44, Lef-1, Frizzled, Tcf(Lef), WNT, Cyclin D1	2.218E-03	3.807E-02	5	NRCAM, Lef-1, ENC1, Tcf(Lef), Cyclin D1
6	IL-6 signaling in multiple myeloma	51	2.435E-06	2.508E-04	2.356E-03	4.935E-02	4	Cyclin D2, Bcl-6, STAT1, Cyclin D1	2.435E-06	2.508E-04	8	STAT3, Cyclin D2, Bcl-6, p53, STAT1, Mcl-1, Cyclin D1, CDK6
7	Fructose metabolism/ Rodent version	85	1.593E-05	1.094E-03	2.413E-01	5.282E-01	2	PFKP, GPI	1.593E-05	1.094E-03	9	PFKP, TPI1, PFKL, ALDOA, HXK2, F264, F263, HXK1, ALDOC
8	Immune response_Oncostatin M signaling via JAK-Stat in mouse cells	20	1.905E-05	1.121E-03	1.301E-03	3.893E-02	3	SOCS3, STAT1, Cyclin D1	1.905E-05	1.121E-03	5	STAT3, SOCS3, TIMP1, STAT1, Cyclin D1
9	Development_Thrombopoetin signaling via JAK-STAT pathway	22	3.146E-05	1.296E-03	8.813E-05	7.385E-03	4	TAP1 (PSF1), SOCS3, STAT1, Cyclin D1	3.146E-05	1.296E-03	5	STAT3, TAP1 (PSF1), SOCS3, STAT1, Cyclin D1
10	Immune response_Oncostatin M signaling via JAK-Stat in human cells	22	3.146E-05	1.296E-03	1.729E-03	4.479E-02	3	SOCS3, STAT1, Cyclin D1	3.146E-05	1.296E-03	5	STAT3, SOCS3, TIMP1, STAT1, Cyclin D1

Table S10 FC values 10nM FTY720-P vs no FTY720-P in Untreated, Mock-treated and HIV- treated cultures

FC absolute value ≥ 1.3 and nominal p value ≤ 0.05

FTY720-P vs no FTY720-P - Unt cultures

Gene	Fold Change	Nominal p value
FANCG	1.4145	0.0039
PAFAH1B3	1.3484	0.0179
LOC391532	1.3066	0.0162
SLC6A8	-1.3042	0.0191
LOC100008588	-1.3257	0.003
NRP1	-1.3316	0.0248
C14ORF4	-1.3368	0.0082
SC5DL	-1.3428	0.0142
IGFBP3	-1.3756	0.0075
KIAA0182	-1.4044	0.0009
TAGLN	-1.4163	0.0021
ID2	-1.4213	0.0361
LOC100133565	-1.4369	0.0228
TPI1	-1.4497	0.0333
ADM	-1.4861	0.0488
C1ORF61	-1.4915	0.0069
ID3	-1.5007	0.0269
ANXA1	-1.5092	0.004
FST	-1.5264	0.0003
ID2	-1.555	0.0162
ID1	-1.5668	0.0038
LOC441763	-1.7835	0.0158

FTY720-P vs no FTY720-P - Mock exposed

Gene	Fold Change	Nominal p value
RPL28	1.3389	0.0482
RPS29	1.3152	0.0396
ATPBD4	1.3041	0.0397
FST	-1.3081	0.0313
SPP1	-1.3109	0.0164
MCART1	-1.3125	0.0031
TMEM158	-1.3182	0.0195
CYP26A1	-1.3211	0.0444
LAMC1	-1.3373	0.01
FKBP14	-1.3568	0.0291
THBS1	-1.3568	0.0436
TAGLN	-1.3842	0.0038
VGF	-1.4073	0.0354
CYP26A1	-1.4175	0.0181
SNF8	-1.4486	0.019
CYP26A1	-1.4546	0.0193
SPOCK1	-1.4547	0.0127
ANXA1	-1.6331	0.0154

FTY720-P vs no FTY720-P - HIV exposed

Gene	Fold Change	Nominal p value
PALM	1.8913	0.0418
DTD1	1.4715	0.0317
LOC401676	1.4537	0.0061
GM2A	1.4405	0.0324
PAFAH1B3	1.4378	0.017
RPL14	1.3958	0.0308
LOC654350	1.3941	0.035
TST	1.3849	0.0216
PTK7	1.3804	0.0209
PSMD3	1.3763	0.0341
THOC5	1.3446	0.0279
LOC100132761	1.3442	0.0399
LOC729500	1.3422	0.0382
SOCS2	1.3282	0.0343
LOC148430	1.3276	0.0473
DRD1IP	1.3213	0.0498
C17ORF61	1.3212	0.0218
HS6ST1	1.3144	0.029
ATP1A1	-1.3008	0.0299
FST	-1.302	0.0147
TKT	-1.3076	0.0233
SF3B3	-1.3108	0.0366
C22ORF27	-1.3189	0.0178
SLC7A5	-1.3222	0.0059
EML4	-1.3278	0.037
RRAGD	-1.3299	0.0238
FAM167A	-1.3329	0.0193
PPP3R1	-1.3406	0.0047
C19ORF60	-1.3445	0.0202
ENC1	-1.3459	0.0118

EEF1B2	-1.3478	0.003
KIFC1	-1.356	0.0036
CHD8	-1.394	0.043
FLJ35801	-1.4083	0.019
HLA-B	-1.4488	0.0226
LMNB1	-1.4908	0.0009
TAF15	-1.5012	0.0333
APP	-1.532	0.0047
AP2B1	-1.5379	0.0243
RBM39	-1.5647	0.0371
TAF15	-1.5945	0.0313
AP2B1	-1.6203	0.0116
LOC100133565	-2.193	0.0363
LOC100008589	-2.9392	0.0227

Table S11 Enrichment analysis report, canonical pathways- 10nM FTY720-P vs no FTY720-P in Untreated, Mock-treated and HIV- treated cultures

Enrichment by Pathway Maps					HIV +10nM FTY720-P vs no FTY720-P				Mock +10nM FTY720-P vs no FTY720-P				Unt +10nM FTY720-P vs no FTY720-P			
#	Maps	Total	min p Value	Min FDR	p-value	FDR	In Data	Network Objects from Active Data	p-value	FDR	In Data	Network Objects from Active Data	p-value	FDR	In Data	Network Objects from Active Data
1	Development_Transcription factors in segregation of hepatocytic lineage	30	2.450E-02	5.777E-02	6.890E-02	1.549E-01	1	Follistatin	2.450E-02	5.777E-02	1	Follistatin	3.654E-02	9.594E-02	1	Follistatin
2	Development_BMP signaling	33	2.693E-02	5.777E-02	7.553E-02	1.549E-01	1	Follistatin	2.693E-02	5.777E-02	1	Follistatin	4.012E-02	9.594E-02	1	Follistatin
3	Signal transduction_Activin A signaling regulation	33	2.693E-02	5.777E-02	7.553E-02	1.549E-01	1	Follistatin	2.693E-02	5.777E-02	1	Follistatin	4.012E-02	9.594E-02	1	Follistatin
4	Reproduction_Gonadotropin-releasing hormone (GnRH) signaling	72	5.793E-02	6.758E-02	1.578E-01	1.726E-01	1	Follistatin	5.793E-02	6.758E-02	1	Follistatin	8.564E-02	1.080E-01	1	Follistatin

Table S12 Enrichment by GO Processes- 10nM FTY720-P vs no FTY720-P in Untreated, Mock-treated and HIV- treated cultures

Enrichment by GO Processes					HIV +10nM FTY720-P vs no FTY720-P				Mock +10nM FTY720-P vs no FTY720-P				Unt +10nM FTY720-P vs no FTY720-P			
#	Processes	Total	min(pValue)	Min FDR	p-value	FDR	In Data	Network Objects from Active Data	p-value	FDR	In Data	Network Objects from Active Data	p-value	FDR	In Data	Network Objects from Active Data
1	innate immune response	956	1.371E-25	1.456E-23	1.371E-25	1.456E-23	31	HLA-B14, HLA-B40, HLA-B49, HLA-B38, HLA-B42, HLA-B55, HLA-B37, HLA-B46, HLA-B58, HLA-B8, HLA-B81, HLA-B, HLA-B54, HLA-B15, HLA-B52, HLA-B41, HLA-B35, 1B73, APP, HLA-B39, MHC Class I alpha chain, HLA-B57, HLA-B44, HLA-B27, HLA-B51, HLA-B53, HLA-B Cw-5, HLA-B78, HLA-B7, HLA-B13, HLA-B18	5.203E-01	5.675E-01	1	Annexin I	5.203E-01	5.419E-01	1	Annexin I
2	response to cytokine	1222	6.733E-25	6.752E-23	6.733E-25	6.752E-23	33	HLA-B14, HLA-B40, SOCS2, HLA-B49, HLA-B38, HLA-B42, HLA-B55, HLA-B37, HLA-B46, HLA-B58, TAFs, HLA-B8, HLA-B81, HLA-B, HLA-B54, HLA-B15, HLA-B52, PSMD3, HLA-B41, HLA-B35, 1B73, HLA-B39, MHC Class I alpha chain, HLA-B57, HLA-B44, HLA-B27, HLA-B51, HLA-B53, HLA-B Cw-5, HLA-B78, HLA-B7, HLA-B13, HLA-B18	2.334E-01	3.200E-01	2	Annexin I, Thrombospondin 1	6.112E-01	6.260E-01	1	Annexin I
3	regulation of immune response	1346	1.421E-23	1.116E-21	1.421E-23	1.116E-21	33	HLA-B14, HLA-B40, HLA-B49, HLA-B38, HLA-B42, HLA-B55, HLA-B37, HLA-B46, HLA-B58, HLA-B8, HLA-B81, Calcineurin B (regulatory), HLA-B, HLA-B54, HLA-B15, Calcineurin B1, HLA-B52, PSMD3, HLA-B41, HLA-B35, 1B73, HLA-B39, MHC Class I alpha chain, HLA-B57, HLA-B44, HLA-B27, HLA-B51, HLA-B53, HLA-B Cw-5, HLA-B78, HLA-B7, HLA-B13, HLA-B18	6.478E-01	6.780E-01	1	Annexin I	6.478E-01	6.595E-01	1	Annexin I
4	cell surface receptor signaling pathway	2970	8.652E-21	6.507E-19	8.652E-21	6.507E-19	41	HLA-B14, PTK7, HLA-B40, SOCS2, HLA-B49, HLA-B38, HLA-B42, DDR2, HLA-B55, HLA-B37, HLA-B46, HLA-B58, Follistatin, HLA-B8, HLA-B81, Calcineurin B (regulatory), HLA-B, HLA-B54, HLA-B15, Calcineurin B1, HLA-B52, PSMD3, HLA-B41, HLA-B35, 1B73, Beta-adaptin 2, APP, ATP1A3, HLA-B39, MHC Class I alpha chain, HLA-B57, HLA-B44, HLA-B27, HLA-B51, HLA-B53, HLA-B Cw-5, HLA-B78, CHD8, HLA-B7, HLA-B13, HLA-B18	3.902E-01	4.562E-01	3	SGPP1, Annexin I, Follistatin	6.198E-02	1.180E-01	5	Neuropilin-1, Annexin I, ID1, Follistatin, IBP

5	cellular response to organic substance	3023	1.686E-20	1.217E-18	1.686E-20	1.217E-18	41	HLA-B14, PTK7, HLA-B40, SOCS2, HLA-B49, RagD, Lamin B1, HLA-B38, HLA-B42, HLA-B55, HLA-B37, HLA-B46, HLA-B58, TAFs, Follistatin, HLA-B8, HLA-B81, HLA-B, HLA-B54, HLA-B15, HLA-B52, Lamin B, PSMD3, HLA-B41, HLA-B35, 1B73, APP, ATP1A3, ATP1A1, HLA-B39, MHC Class I alpha chain, HLA-B57, HLA-B44, HLA-B27, HLA-B51, HLA-B53, HLA-B Cw-5, HLA-B78, HLA-B7, HLA-B13, HLA-B18	1.886E-02	9.967E-02	6	CYP26A1, CXXC1, Annexin I, Thrombospondin 1, FKBP14, Follistatin	1.886E-02	6.390E-02	6	Neuropilin-1, Annexin I, ID3, ID1, Follistatin, IBP
6	regulation of immune system process	2131	1.712E-19	1.189E-17	1.712E-19	1.189E-17	35	HLA-B14, HLA-B40, HLA-B49, HLA-B38, HLA-B42, HLA-B55, HLA-B37, HLA-B46, HLA-B58, HLA-B8, HLA-B81, Calcineurin B (regulatory), HLA-B, HLA-B54, HLA-B15, Calcineurin B1, HLA-B52, PSMD3, FMIP, HLA-B41, HLA-B35, 1B73, Beta-adaptin 2, HLA-B39, MHC Class I alpha chain, HLA-B57, HLA-B44, HLA-B27, HLA-B51, HLA-B53, HLA-B Cw-5, HLA-B78, HLA-B7, HLA-B13, HLA-B18	4.854E-01	5.381E-01	2	Annexin I, Thrombospondin 1	2.116E-01	2.579E-01	3	Annexin I, ID2, IBP
7	immune response	1680	2.165E-18	1.447E-16	2.165E-18	1.447E-16	31	HLA-B14, HLA-B40, HLA-B49, HLA-B38, HLA-B42, HLA-B55, HLA-B37, HLA-B46, HLA-B58, HLA-B8, HLA-B81, HLA-B, HLA-B54, HLA-B15, HLA-B52, HLA-B41, HLA-B35, 1B73, APP, HLA-B39, MHC Class I alpha chain, HLA-B57, HLA-B44, HLA-B27, HLA-B51, HLA-B53, HLA-B Cw-5, HLA-B78, HLA-B7, HLA-B13, HLA-B18	3.636E-01	4.336E-01	2	Annexin I, Thrombospondin 1	3.636E-01	3.979E-01	2	Annexin I, Adrenomedullin
8	defense response	1948	1.422E-17	9.169E-16	1.422E-17	9.169E-16	32	HLA-B14, HLA-B40, HLA-B49, HLA-B38, HLA-B42, HLA-B55, HLA-B37, HLA-B46, HLA-B58, HLA-B8, HLA-B81, Calcineurin B (regulatory), HLA-B, HLA-B54, HLA-B15, HLA-B52, HLA-B41, HLA-B35, 1B73, APP, HLA-B39, MHC Class I alpha chain, HLA-B57, HLA-B44, HLA-B27, HLA-B51, HLA-B53, HLA-B Cw-5, HLA-B78, HLA-B7, HLA-B13, HLA-B18	5.271E-02	1.480E-01	4	VGF, Osteopontin, Annexin I, Thrombospondin 1	1.759E-01	2.241E-01	3	Annexin I, Adrenomedullin, IBP

9	cellular response to chemical stimulus	3629	1.521E-17	9.470E-16	1.521E-17	9.470E-16	41	HLA-B14, PTK7, HLA-B40, SOCS2, HLA-B49, RagD, Lamin B1, HLA-B38, HLA-B42, HLA-B55, HLA-B37, HLA-B46, HLA-B58, TAFs, Follistatin, HLA-B8, HLA-B81, HLA-B, HLA-B54, HLA-B15, HLA-B52, Lamin B, PSMD3, HLA-B41, HLA-B35, 1B73, APP, ATP1A3, ATP1A1, HLA-B39, MHC Class I alpha chain, HLA-B57, HLA-B44, HLA-B27, HLA-B51, HLA-B53, HLA-B Cw-5, HLA-B78, HLA-B7, HLA-B13, HLA-B18	1.195E-02	9.061E-02	7	CYP26A1, Osteopontin, CXXC1, Annexin I, Thrombospondin 1, FKBP14, Follistatin	1.195E-02	5.179E-02	7	Neuropilin-1, Annexin I, ID3, ID2, ID1, Follistatin, IBP
10	response to organic substance	4151	2.554E-16	1.537E-14	2.554E-16	1.537E-14	42	HLA-B14, PTK7, HLA-B40, SOCS2, HLA-B49, RagD, Lamin B1, HLA-B38, HLA-B42, HLA-B55, HLA-B37, HLA-B46, HLA-B58, TAFs, Follistatin, HLA-B8, HLA-B81, HLA-B, eEF1B, HLA-B54, HLA-B15, HLA-B52, Lamin B, PSMD3, HLA-B41, HLA-B35, 1B73, APP, ATP1A3, ATP1A1, HLA-B39, MHC Class I alpha chain, HLA-B57, HLA-B44, HLA-B27, HLA-B51, HLA-B53, HLA-B Cw-5, HLA-B78, HLA-B7, HLA-B13, HLA-B18	6.403E-03	8.033E-02	8	VGF, CYP26A1, Osteopontin, CXXC1, Annexin I, Thrombospondin 1, FKBP14, Follistatin	2.435E-02	7.124E-02	7	Neuropilin-1, Annexin I, ID3, ID1, Adrenomedullin, Follistatin, IBP
